# Unearthing a fungal giant: *Dianjunaceae* fam. nov., a novel Paleocene lineage of *Xylariales* harboring *Dianjunus rex* gen. et sp. nov

**DOI:** 10.3389/ffunb.2026.1940464

**Published:** 2026-09-29

**Authors:** Jing Song, Zhuyue Yan, Jesús Pérez-Moreno, Fengming Zhang, Shimei Yang, Tao Xie, Luofeng Su, Jianwei Liu, Yanliang Wang, Dong Liu, Zhenyan Yang, Chengmo Yang, Wei Liu, Xiaofei Shi, Shanping Wan, Ratchadawan Cheewangkoon, Dongqin Dai, Indunil C. Senanayake, Fuqiang Yu

**Affiliations:** 1 Yunnan Key Laboratory for Fungal Diversity and Green Development and Yunnan International Joint Laboratory of Fungal Sustainable Utilization in South and Southeast Asia, Germplasm Bank of Wild Species, Kunming Institute of Botany, Chinese Academy of Sciences, Kunming, Yunnan, China; 2 Departments of Plant Pathology and Entomology, Faculty of Agriculture, Chiang Mai University, Chiang Mai, Thailand; 3 Campus Montecillo, Colegio de Postgraduados, Texcoco de Mora, Mexico; 4 Key Laboratory of Chemistry in Ethnic Medicinal Resources, School of Ethnic Medicine, Yunnan Minzu University, Kunming, Yunnan, China; 5 College of Resources and Environment, Yunnan Agricultural University, Kunming, Yunnan, China; 6 Center for Yunnan Plateau Biological Resources Protection and Utilization and Yunnan International Joint Laboratory of Fungal Sustainable Utilization in South and Southeast Asia, College of Biology and Food Engineering, Qujing Normal University, Qujing, Yunnan, China

**Keywords:** divergence time, genome sequences, large stromata, morphology, novel taxa

## Abstract

During mycological surveys in Yunnan Province, China, specimens of a fungus producing massive, upright stromata up to 50 cm high and individually 2.2 kg in weight were sampled. Through an integrative taxonomic approach combining detailed morphology, multilocus phylogeny [internal transcribed spacer (ITS), large subunit ribosomal DNA (LSU), RNA polymerase II subunit 2 (*RPB2)*, and beta-tubulin (*TUB2)*], and phylogenomic analyses, this fungus is proposed as the new species *Dianjunus rex* gen. et sp. nov., the type of the new family *Dianjunaceae* (*Xylariales*). Phylogenetic analyses robustly place *Dianjunaceae* as a distinct sister clade to *Graphostromataceae*. Divergence time estimation dates the origin of this family to the early Paleocene (~65 million years ago), coinciding with the period following the Cretaceous–Paleogene (K–Pg) extinction, when an estimated 75% of all plant and animal species went extinct, and a significant ecological reorganization of life on Earth happened. The stromata of *D. rex* represent the largest fructifications documented within the *Ascomycota*, significantly expanding the known morphological range of the *Xylariales*. The study provides a comprehensive description, including a nodulisporium-like anamorph with periconiella-like branching patterns, and discusses the taxon’s phylogenetic placement and distinctive morphology. This discovery highlights the unexplored fungal diversity in East Asian forests.

## 1 Introduction

The order *Xylariales* Nannf. comprises a morphologically diverse group of ascomycetous fungi, typically characterized by carbonaceous, darkly pigmented stromata. Taxa within this order occupy varied ecological niches, functioning as endophytes, pathogens, or saprobes (Senanayake et al., 2015). Saprobic members, often wood-associated, are key decomposers of dead plant materials, particularly lignified tissues, and contribute significantly to carbon cycling in forest ecosystems (Ma et al., 2022). Endophytic xylarialean species reside asymptomatically within healthy plant tissues, sometimes shifting to saprobic life mode when host tissues senesce, while others may act as opportunistic plant pathogens under host stress (Cedeño-Sanchez et al., 2023).

*Xylariales* typically produce perithecial ascomata embedded within stromatic tissues. They are defined by unitunicate asci, frequently possessing a characteristic amyloid or inamyloid apical apparatus, and brown to dark ascospores that often exhibit a germ-slit (Stadler, 2011). While traditional taxonomy has relied on stromatal morphology, perithecial arrangement, and spore characteristics, these traits are often subject to convergence. Contemporary systematics of *Xylariales* increasingly integrates multi-locus phylogenetic and genome-scale data, leading to substantial revisions of familial and generic boundaries (Wendt et al., 2018; Chen et al., 2023). Several studies indicated that anamorph morphology in *Xylariales* fungi served as a better indicator of phylogenetic relationships than teleomorph morphology (Jaklitsch and Voglmayr, 2012; Jaklitsch et al., 2016). The order currently encompasses 16 recognized families (Samarakoon et al., 2016) and allied lineages reflecting considerable lifestyle diversity. Beyond their ecological relevance, *Xylariales* are notable for their prolific production of secondary metabolites, including pigments and bioactive compounds, and their enzymatic capacity to degrade complex plant polymers. These attributes render them of significant interest for biotechnology, natural product discovery, and studies of plant–microbe interaction (Becker and Stadler, 2021; Franco et al., 2025).

Stromatal morphology within the order varies, including both upright and penzigioid forms (Fournier et al., 2020). The order *Xylariales* encompasses few species capable of producing large stromata. A paramount example is *Engleromyces goetzei* P. Henn., which forms large, subglobose stromata on bamboo culms (Whalley et al., 2010). A documented specimen from Kenya comprised two lobes measuring 30 cm in diameter with a mass of approximately 3.2 kg, ranking among the most voluminous individual ascomata recorded in *Ascomycota* (Gibson and Kimeria, 1962; Whalley et al., 2010). In contrast, species such as *Xylaria polymorpha* (Pers.) Grev. and *X. longipes* Nitschke produce clustered, clavate stromata where aggregations, though comprised of modest individual units (5–8 cm height), can exceed 20 cm in total breadth (Rogers, 1979). *Squamotubera le-ratii* Henn., the only species of the genus, has been reported with stromata around 7 cm height and 5 cm width (Hennings, 1903; Rogers, 1981).

During fungal diversity surveys in Yunnan Province, China, several specimens producing exceptionally large, upright stromata were collected from a tropical rainforest in Xishuangbanna Dai Autonomous Prefecture and from temperate coniferous forests in Kunming and Lufeng Cities. Through integrative approaches combining detailed morphological examination with multilocus phylogenetic reconstruction, taxonomic placement of all specimens was determined.

## 2 Materials and methods

### 2.1 Sample collection and morphological examination

Specimens were collected during the following periods and in the following locations: August to September 2024 in Damaidi Village, Tuanjie Town, Luquan County, Kunming, China; July 2025 in Nanbang Village, Mengla County, Xishuangbanna Dai Autonomous Prefecture, Yunnan Province, China; and July 2025 in Yipinglang Town, Lufeng City, Chuxiong Yi Autonomous Prefecture, Yunnan Province, China. Detailed site data, including elevation, climatic conditions, and geographical features, were recorded *in situ*. Photographs of fungal specimens in their natural habitat were taken using a Canon G15 camera (Canon Inc., Tokyo, Japan). Collected materials were wrapped in waterproof foil and transported to the laboratory for subsequent analysis. Macroscopic characteristics were examined using an Olympus SZ61 stereomicroscope. Micromorphological structures, including ascospores, asci, and ascomata, were photographed using a Canon 700D digital camera mounted on a compound microscope. Ascospores were cultured on Potato Dextrose Agar (PDA), and pure cultures were obtained following the methodology of Senanayake et al. (2020b). Fungal hyphae were grown on Malt extract agar (MEA), Oat Meal Agar (OMA), and PDA to examine the colony morphology. All microscopic measurements were performed using the Tarosoft Image Frame Work (v. 0.9.0.7). Image composites were processed and adjusted with Adobe Photoshop CS6 software (version 10.0, Adobe Systems, USA). Voucher specimens were deposited at the fungarium of the Kunming Institute of Botany, Chinese Academy of Sciences (HKAS). Index Fungorum numbers (http://www.indexfungorum.org) were registered for all novel taxa.

### 2.2 DNA extraction, PCR amplification, and sequencing

Genomic DNA was extracted from the fresh stromata tissue using the Biospin DNA Extraction Kit (Hangzhou Bioer Technology Co., Ltd., Hangzhou, China) according to the manufacturer’s protocol. Extracted DNA was stored at -20 °C before amplification. Polymerase chain reaction (PCR) amplification and sequencing were performed for four genetic loci: the partial 28S Large Subunit ribosomal DNA (LSU) using primers LR0R and LR5 (Vilgalys and Hester, 1990); the complete Internal Transcribed Spacer (ITS) region using primers ITS1 and ITS4 (White et al., 1990); the beta-tubulin (*TUB2*) gene using primers T1 and T22 (O'Donnell and Cigelnik, 1997); and the RNA polymerase II subunit 2 (RPB2) gene using primers fRPB2-5F and fRPB2-7cR (Liu et al., 1999). PCR reactions were conducted following the protocol described in Senanayake et al. (2020b). The total volume of the PCR reaction was 25 µL, containing 1 µL of DNA template, 1 µL of each forward and reverse primer, 12.5 µL of 2 × PCR Master Mix, and 9.5 µL of sterile double-distilled water (ddH_2_O). Amplification was performed for 35 cycles under the thermocycling conditions detailed in **Table 1**. PCR products were visualized via electrophoresis on 1% agarose gels stained with ethidium bromide. Products were purified and sequenced commercially (Sunbiotech Company, Beijing, China). Raw sequences were assembled, edited, and quality-checked using DNASTAR Lasergene v. 7.1. Newly generated sequences were deposited in the National Center for Biotechnology Information (NCBI)GenBank database; corresponding accession numbers are provided in **Table 2**.

**Table 1:** Primers and optimized PCR protocols used in this study.

Locus	PCR protocols (Annealing temperature in bold)	References
LSU	(94 °C: 1 min, **56 °C**: 50 s, 72 °C: 1 min) × 35 cycles	Vilgalys and Hester (1990)
ITS	(94 °C: 50 s, **56 °C**: 50 s, 72 °C: 1 min) × 35 cycles	White et al. (1990)
TUB2	(94 °C: 30 s, **57 °C**: 50 s, 72 °C: 1 min) × 35 cycles	O'Donnell and Cigelnik (1997)
RPB2	(95 °C: 30 s, **55 °C**: 50 s, 72 °C: 1 min) × 35 cycles	Liu et al. (1999)

**Table 2:** Details of the isolates used in the phylogenetic tree.

Species	Culture accession number	ITS	LSU	*RPB2*	*TUB2*	References
*Acrocordiella omanensis*	SQUCC 15091^T^	MG584568	MG584570	−	−	Maharachchikumbura et al. (2018)
*Acrocordiella yunnanensis*	HKAS 111922^T^	MW424507	MW424505	−	−	Dissanayake et al. (2021)
*Alloanthostomella rubicola*	MFLUCC 16-0479	KX533455	KX533456	−	−	Daranagama et al. (2015)
*Allocryptovalsa cryptovalsoidea*	HVFIG02	HQ692573	−	−	HQ692524	Trouillas et al. (2011)
*Allocryptovalsa elaeidis*	MFLUCC 15-0707^T^	MN308410	MN308401	−	MN340296	Konta et al. (2020)
*Allocryptovalsa microtheca*	MFLUCC 18-1434	PV617424	PV617572	PV902086	PV901980	Dissanayake et al. (2025)
*Allocryptovalsa polyspora*	MFLUCC 17-0364^T^	MF959500	MF959503	−	MG334556	Senwanna et al. (2017)
*Allocryptovalsa rabenhorstii*	WA08CB	HQ692619	−	−	HQ692523	Senwanna et al. (2017)
*Allocryptovalsa truncate*	PUFNI17639^T^	MK990279	MK981538	−	−	Hyde et al. (2020a)
*Allodiatrype arengae*	MFLUCC 15-0713^T^	MN308411	MN308402	MN542886	MN340297	Konta et al. (2020)
*Alloeutypa flavovirens*	CHUNI6	KR092798	KR092774	−	−	Senanayake et al. (2015)
*Amphibambusa bambusicola*	MFLUCC 11-0617^T^	KP744433	KP744474	−	−	Liu et al. (2015)
*Amphirosellinia nigrospora*	HAST 91092308^T^	GU322457	−	GQ848340	GQ495951	Hsieh et al. (2010)
*Annulohypoxylon terebratum*	CBS 119137^T^	DQ631943	DQ840069	DQ631954	DQ840097	Fournier et al. (2010)
*Annulohypoxylon truncatum*	CBS 140778^T^	KY610419	KY610419	KY624277	KX376352	Wendt et al. (2018)
*Anthostoma decipiens*	CD	KC774565	KC774565	−	−	Jaklitsch et al. (2014)
*Anthostomella obesa*	MFLUCC 14-0171^T^	KP297405	KP340546	KP340533	KP406616	Daranagama et al. (2015)
*Anthostomelloides krabiensis*	MFLUCC 15-0678	KX305927	KX305928	KX305929	−	Tibpromma et al. (2017)
*Arecophila miscanthi*	FU31025	MK503821	MK503827	−	−	Karunarathna et al. (2021)
*Ascotricha chartarum*	CBS 902.69	MH859477	MH871257	−	−	Vu et al. (2019)
*Ascotricha erinacea*	CBS 535.73	KF893285	MH872482	−	KF893272	Cheng et al. (2015)
*Ascotricha rugispora*	NBRC 33167^T^	LC146763	LC146763	−	−	Okane et al. (2001)
*Astrocystis mirabilis*	HAST 94070803	GU322448	−	GQ844835	GQ495941	Hsieh et al. (2010)
*Barrmaelia macrospora*	CBS 142768^T^	KC774566	KC774566	MF488995	MF489014	Jaklitsch et al. (2014)
*Barrmaelia rappazii*	CBS 142771^T^	MF488989	MF488989	MF488998	MF489017	Voglmayr et al. (2018)
*Barrmaelia rhamnicola*	CBS 142772^T^	MF488990	MF488990	MF488999	MF489018	Voglmayr et al. (2018)
*Biscogniauxia anceps*	YMJ 123	EF026132	−	JX507777	AY951671	Hsieh et al. (2010)
*Biscogniauxia capnodes*	YMJ 138	EF026131	−	JX507779	AY951675	Hsieh et al. (2010)
*Biscogniauxia mangiferae*	MFLU 18-0827^T^	MN337232	MN336236	MN366247	MN509782	Hyde et al. (2020a)
*Biscogniauxia nummularia*	MUCL 51395^T^	KY610382	KY610427	KY624236	KX271241	Wendt et al. (2018)
*Brunneiperidium gracilentum*	MFLUCC 14-0011^T^	KP297400	KP340542	KP340528	KP406611	Daranagama et al. (2015)
*Cainia graminis*	MFLUCC 15-0540	KR092793	KR092781	−	−	Senanayake et al. (2015)
*Calceomyces lacunosus*	CBS 633.88^T^	KY610397	KY610476	KY624293	KX271265	Wendt et al. (2018)
*Camillea obularia*	ATCC 28093	KY610384	KY610429	KY624238	KX271243	Wendt et al. (2018)
*Castellaniomyces rosae*	MFLUCC 15-0536^T^	MF614127	MF614130	−	−	Senanayake et al. (2015)
*Circinotrichum maculiforme*	CBS 122758	KR611875	KR611896	−	−	Crous et al. (2015)
*Clypeosphaeria mamillana*	CBS140735^T^	KT949897	KT949897	MF489001	MH704637	Jaklitsch et al. (2016)
*Clypeosphaeria mamillana*	WU 33599	KT949898	KT949898	−	−	Jaklitsch et al. (2016)
*Clypeosphaeria mamillana*	WU 36850	KT949899	KT949899	−	−	Jaklitsch et al. (2016)
*Clypeosphaeria perfidiosa*	CBS 142773	MF488993	−	MF489003	MF489021	Vu et al. (2019)
*Collodiscula japonica*	CBS 124266	JF440974	MH874889	KY624273	−	Jaklitsch and Voglmayr (2012)
*Coniocessia anandra*	CBS 125766^T^	MH863747	MH875215	−	−	Vu et al. (2019)
*Coniocessia cruciformis*	CBS 126674	−	MH875663	−	−	Vu et al. (2019)
*Coniocessia maxima*	CBS 593.74^T^	GU553332	MH878275	−	−	Vu et al. (2019)
*Coniocessia nodulisporioides*	CBS 281.77^T^	MH861061	MH872831	−	−	Vu et al. (2019)
*Coniolariella gamsii*	Co27	GU553325	GU553329	−	−	Vu et al. (2019)
*Creosphaeria sassafras*	STMA 14087	KY610411	KY610468	KY624265	KX271258	Wendt et al. (2018)
*Cryptosphaeria eunomia*	CBS216.87^T^	KT425230	KT425296	−	−	Trouillas et al. (2015)
*Cryptosphaeria subcutanea*	CBS 240.87^T^	KT425232	KT425297	−	−	Trouillas et al. (2015)
*Cryptostroma corticale*	CBS 216.52	HG934110	MH868529	HG934116	HG934102	Vu et al. (2019)
*Cryptovalsa ampelina*	ATCC MYA-4399	FJ430591	FJ430582	−	−	Trouillas et al. (2010)
*Daldinia concentrica*	CBS 113277	AY616683	KY610434	KY624243	KC977274	Triebel et al. (2005)
*Daldinia loculatoides*	CBS 113279^T^	AF176982	KY610438	KY624247	KX271246	Triebel et al. (2005)
*Daldinia* sp.	ATCC 46302	KY610389	KY610443	KY624253	KX271248	Wendt et al. (2018)
*Delonicicola siamense*	MFLUCC 15-0670^T^	MF167586	MF158345	MF158346	−	Perera et al. (2017)
*Dematophora bunodes*	CBS 124028	MN984619	MN984625	−	MN987245	Wittstein et al. (2020)
*Dematophora necatrix*	89062904	EF026117	−	−	EF025603	Hsieh et al. (2010)
*Dematophora necatrix*	CBS 349.36	AY909001	KF719204	KY624275	KY624310	Peláez et al. (2008)
*Diabolocovidia claustri*	CPC37593^T^	MT373367	MT373350	−	−	Crous et al. (2020)
***Dianjunus rex***	**HKAS148941^T^**	**PX777991**	**PX777996**	**PX987258**	**PX930195**	**This study**
***Dianjunus rex***	**HKAS148942**	**PX777992**	**PX777997**	**PX987259**	**PX930196**	**This study**
***Dianjunus rex***	**HKAS150823**	**PX777993**	**PX777998**	**PX987260**	**−**	**This study**
***Dianjunus rex***	**HKAS150824**	**PX777994**	**PX777999**	**PX987261**	**PX930197**	**This study**
***Dianjunus rex***	**HKAS151626**	**PX777995**	**PX778000**	**PX987262**	**−**	**This study**
*Diatrype bullata*	UCDDCH400	DQ006946	−	−	DQ007002	Rolshausen et al. (2006)
*Diatrype disciformis*	AFTOL-ID 927	DQ470964	U47829	DQ470915	−	Spatafora et al. (2006)
*Diatrype lijiangensis*	MFLU 19-0717^T^	MK852582	MK810546	−	MK852583	Thiyagaraja et al. (2019)
*Diatrype virescens*	CBS 128344	MH864890	MH876339	−	−	Vu et al. (2019)
*Diatrypella favacea*	CBS 198.49	MH856491	MH868027	−	−	Vu et al. (2019)
*Diatrypella heveae*	MFLUCC 15-0274^T^	MN308418	MN308409	MN542892	MN340301	Vu et al. (2019)
*Diatrypella tectonae*	MFLUCC 12-0172b^T^	KY283085	KY283080	−	−	Shang et al. (2017)
*Didymobotryum rigidum*	JCM 8837^T^	LC228650	LC228707	−	−	Schoch et al. (2012)
*Discoxylaria myrmecophila*	169	GU322433	−	GQ844819	GQ487710	Hsieh et al. (2010)
*Durotheca crateriformis*	GMBC 0205^T^	MH645426	MH645425	MH645427	MH049441	Long et al. (2019)
*Emarcea castanopsidicola*	CBS 117105^T^	AY603496	MK762717	MK791285	MK776962	Duong et al. (2004)
*Endocalyx cinctus*	JCM 7946	LC228648	LC228704	−	−	Schoch et al. (2012)
*Endocalyx metroxyli*	MFLUCC 15-0723A^T^	MT929162	MT929313	−	−	Thitla et al. (2025)
*Engleromyces sinensis*	BJTC 200802^ET^	MZ622704	MZ622701	MZ622185	−	Gao et al. (2023)
*Engleromyces sinensis*	BJTC 200801	MZ622703	MZ622700	MZ622184	MZ622187	Gao et al. (2023)
*Entalbostroma erumpens*	ICMP 21152^T^	KX258206	−	KX258204	KX258205	Johnston et al. (2016)
*Entosordaria quercina*	CBS 142774^T^	MF488994	MF488994	MF489004	MF489022	Voglmayr et al. (2018)
*Eutypa lata*	DSC100	HM164729	−	HM164797	HM164763	Trouillas and Gubler (2010)
*Eutypella quaternata*	MFLU 15-2605	MT185553	MT183518	MT432247	−	Li et al. (2020)
*Fasciatispora arengae*	MFLUCC 15-0326a^T^	MK120275	MK120300	MK890794	MK890793	Doilom et al. (2018)
*Fasciatispora cocoes*	MFLUCC 18-1445^T^	MN482680	MN482675	MN481517	MN505154	Hyde et al. (2020b)
*Fasciatispora nypae*	MFLUCC 11-0382^T^	NR_185352	NG_067532	−	−	Liu et al. (2015)
*Fusiformiascus pulvinatus*	H048	FR715523	FR715523	−	FR715495	Pažoutová et al. (2012)
*Graphostroma platystomum*	CBS 270.87^T^	JX658535	DQ836906	KY624296	HG934108	Stadler et al. (2014)
*Guayaquilia cubensis*	MUCL 39017	KC775733	KC775708	−	−	Becerra-Hernández et al. (2016)
*Gyrothrix inops*	BE108	KC775746	KC775721	−	−	Becerra-Hernández et al. (2016)
*Gyrothrix ramose*	MUCL 54061	KC775747	KC775722	−	−	Becerra-Hernández et al. (2016)
*Halocryptovalsa salicorniae*	MFLUCC 15-0185^T^	MH304410	MH305304	−	MH370274	Dayarathne et al. (2020)
*Halodiatrype avicenniae*	MFLUCC 15-0948	MH304414	MH305308	−	MH370278	Dayarathne et al. (2020)
*Halorosellinia krabiensis*	MFLU 17-2596^T^	MN047119	MN017883	−	MN431493	Dayarathne et al. (2020)
*Hansfordia pruni*	CBS 194.56^T^	MK442585	MH869122	KU684307	−	Crous et al. (2019)
*Hansfordia pulvinata*	CBS 144422	MK442587	MK442527	−	−	Crous et al. (2019)
*Hansfordia pulvinata*	CBS 254.59	KF893288	MH869394	−	−	Crous et al. (2019)
*Haploanthostomella elaeidis*	MFLU 20-0522^T^	MT929161	MT929312	−	−	Thitla et al. (2025)
*Hypocopra anomala*	TTI-000339	−	MT903245	MT901033	MT901030	Becker et al. (2020)
*Hypocopra dolichopoda*	TTI-000310	−	MT903247	MT901035	−	Becker et al. (2020)
*Hypocopra rostrata*	NRRL 66178	KM067909	KM067909	−	−	Jayanetti et al. (2014)
*Hypomontagnella monticulosa*	MFLUCC 18-0362	MN337231	MN336235	MN366246	MN509783	Lambert et al. (2019)
*Hypoxylon fragiforme*	MUCL 51264^T^	KC477229	KM186295	KM186296	KX271282	Daranagama et al. (2015)
*Hypoxylon lienhwacheense*	MFLUCC 14-1231	KU604558	MK287550	MK287563	KU159522	Sir et al. (2019)
*Pyrenopolyporus hunteri*	STMA07009	−	−	KY624309	−	Wendt et al. (2018)
*Idriella lunata*	CBS 204.56^T^	MH857584	MH869129	−	−	Vu et al. (2019)
*Jackrogersella multiformis*	CBS 119016^T^	KC477234	KY610473	KY624290	KX271262	Wendt et al. (2018)
*Kretzschmaria deusta*	CBS163.93	−	−	KY624227	−	Wendt et al. (2018)
*Kretzschmariella culmorum*	JDR88	KX430043	−	KX430045	KX430046	Fournier et al. (2018)
*Leptosillia cordylines*	MFLU 20-0056	MT073349	NG_068699	MT023134	MT023136	Senanayake et al. (2020a)
*Linosporopsis ischnotheca*	LIF1	MN818952	MN818952	MN820708	MN820715	Voglmayr and Beenken (2020)
*Linosporopsis ochracea*	LIO3	MN818958	MN818958	MN820714	MN820721	Voglmayr and Beenken (2020)
*Longiappendispora chromolaenae*	MFLU 20-0320^T^	MT214370	MT214464	−	−	Mapook et al. (2020)
*Lopadostoma americanum*	CBS 133211^T^	KC774568	KC774568	KC774525	−	Jaklitsch et al. (2014)
*Lopadostoma dryophilum*	LG21	NR_132028	KC774570	KC774526	MF489023	Jaklitsch et al. (2014)
*Lopadostoma fagi*	CBS 133206^T^	KC774575	KC774575	KC774531	−	Jaklitsch et al. (2014)
*Lopadostoma gastrinum*	MFLU 17-0941	MN337229	MN336233	MN366245	−	Jaklitsch et al. (2014)
*Lopadostoma insulare*	CBS 133214^T^	KC774589	KC774589	KC774542	−	Jaklitsch et al. (2014)
*Lopadostoma lechatii*	CBS 133694^T^	KC774590	KC774590	KC774543	−	Jaklitsch et al. (2014)
*Lopadostoma quercicola*	CBS 133212^T^	KC774610	KC774610	KC774558	−	Jaklitsch et al. (2014)
*Lopadostoma turgidum*	CBS 133207^T^	KC774618	KC774618	KC774563	MF489024	Jaklitsch et al. (2014)
*Lunatiannulus irregularis*	MFLUCC 14-0014^T^	KP297398	KP340540	KP340526	KP406609	Daranagama et al. (2015)
*Melanographium phoenicis*	MFLUCC 18-1481^T^	MN482677	MN482678	−	−	Hyde et al. (2020a)
*Microdochium lycopodinum*	CBS 125585^T^	JF440979	JF440979	KP859125	KP859080	Jaklitsch and Voglmayr (2012)
*Microdochium phragmitis*	CBS 285.71^T^	KP859013	KP858949	KP859122	KP859077	Jaklitsch and Voglmayr (2012)
*Monosporascus cannonballus*	MC1703	JQ743053	−	−	JQ804936	Cavalcante et al. (2020)
*Muscodor thailandicus*	MFLUCC 17-2669^T^	MK762707	MK762714	MK791283	MK776960	Samarakoon et al. (2020)
*Muscodor ziziphi*	MFLUCC 17-2662	MK762705	MK762712	MK791281	MK776958	Samarakoon et al. (2020)
*Nemania abortive*	BISH467^T^	GU292816	−	GQ844768	GQ470219	Hsieh et al. (2010)
*Nemania beaumontii*	HAST405	GU292819	−	GQ844772	GQ470222	Hsieh et al. (2010)
*Nemania bipapillata*	HAST90080610	GU292818	−	GQ844771	GQ470221	Hsieh et al. (2010)
*Nemania diffusa*	HAST91020401	GU292817	−	GQ844769	GQ470220	Hsieh et al. (2010)
*Nemania illita*	YMJ236	EF026122	−	GQ844770	EF025608	Hsieh et al. (2010)
*Nemania macrocarpa*	WSP265^T^	GU292823	MH874423	GQ844776	GQ470226	Hsieh et al. (2010)
*Nemania maritima*	HAST89120401^T^	GU292822	−	GQ844775	GQ470225	Hsieh et al. (2010)
*Nemania primolutea*	YMJ91102001^T^	EF026121	−	GQ844767	EF025607	Hsieh et al. (2010)
*Nemania* sp.	BCC30850	KX037428	KX037429	KX037430	−	Kornsakulkarn et al. (2017)
*Nemania sphaeriostoma*	JDR 261	GU292821	−	GQ844774	GQ470224	Hsieh et al. (2010)
*Neoanthostomella fici*	MFLU 19-2765^T^	MW114390	MW114445	MW177711	−	Tennakoon et al. (2021)
*Neoanthostomella viticola*	MFLUCC 16-0243^T^	KX505957	KX505958	KX789496	KX789495	Daranagama et al. (2015)
*Neoeutypella baoshanensis*	MFLUCC 16-1002^T^	MT310662	MT214618	−	−	Phukhamsakda et al. (2020)
*Neoidriella desertorum*	CBS 985.72^T^	KP859048	KP858985	−	−	Hernández-Restrepo et al. (2016)
*Obolarina persica*	YMJ 1461	JX507807	−	JX507792	JX507796	Mirabolfathy et al. (2013)
*Paraeutypella citricola*	HUEFS 194248	KM396616	KM407630	−	KR534602	de Almeida et al. (2016)
*Paraeutypella vitis*	UCD2428TX	FJ790851	−	−	GU294726	Úrbez-Torres et al. (2009)
*Paraxylaria rosacearum*	TASM 6132^T^	MG828941	MG829050	−	−	Wanasinghe et al. (2018)
*Pedumispora rhizophorae*	BCC44877	KJ888853	KJ888850	−	−	Klaysuban et al. (2014)
*Peroneutypa diminutiasca*	MFLUCC 17-2144^T^	MG873479	MG873473	−	MH316765	Shang et al. (2018)
*Peroneutypa diminutispora*	HUEFS 192196^T^	KM396647	KM407664	−	−	de Almeida et al. (2016)
*Peroneutypa mackenziei*	MFLUCC 16-0072^T^	KY283083	KY283079	−	KY706363	Shang et al. (2017)
*Peroneutypa mangrovei*	PUFD526^T^	MG844286	MG844278	−	MH094409	Phookamsak et al. (2019)
*Peroneutypa scoparia*	MFLUCC 11-0615	KU940152	KU863140	KU940180	−	Dai et al. (2016)
*Pirozynskiomyces sinensis*	2184417	KY994106	KY994107	−	−	Li et al. (2017)
*Podosordaria mexicana*	WSP176	GU324762	−	GQ853039	GQ844840	Hsieh et al. (2010)
*Poronia punctate*	CBS 656.78^T^	KT281904	KY610496	KY624278	KX271281	Senanayake et al. (2015)
*Pseudoanthostomella conorum*	CBS 119333	EU552099	EU552099	−	−	Marincowitz et al. (2008)
*Pseudoanthostomella delitescens*	MFLUCC 16-0477	KX533451	KX533452	KX789491	KX789490	Daranagama et al. (2015)
*Pseudoanthostomella pini-nigrae*	MFLUCC 16-0478^T^	KX533453	KX533454	KX789492	−	Daranagama et al. (2015)
*Pseudoceratocladium polysetosum*	FMR10750	KY853430	KY853490	−	−	Hernández-Restrepo et al. (2017)
*Pseudocircinotrichum papakurae*	CBS101373	KR611876	KR611897	−	−	Crous et al. (2015)
*Pyrenomyxa morganii*	CBS 118186^T^	MH863028	MH874581	−	−	Vu et al. (2019)
*Pyrenopolyporus hunteri*	MUCL 52673^T^	KY610421	KY610472	KY624309	KU159530	Wendt et al. (2018)
*Pyriformiascoma trilobatum*	MFLUCC 14-0012^T^	KP297402	KP340543	KP340530	KP406613	Daranagama et al. (2015)
*Requienella fraxini*	CBS 140475^T^	NR_138415	KT949910	−	−	Jaklitsch et al. (2016)
*Requienella seminuda*	CBS 140502^T^	NR_154630	KT949912	MK523300	−	Jaklitsch et al. (2016)
*Rhopalostroma angolense*	CBS 126414	FN821965	KM186298	KM186297	KM186299	Stadler et al. (2010)
*Rosellinia aquila*	MUCL 51703	KY610392	KY610460	KY624285	KX271253	Wendt et al. (2018)
*Rosellinia chiangmaiensis*	MFLUCC 15-0015^T^	KU246226	KU246227	−	−	Daranagama et al. (2015)
*Rosellinia corticium*	MUCL 51693	KY610393	KY610461	KY624229	KX271254	Wendt et al. (2018)
*Rosellinia limonispora*	CBS 382.86	KR611876	KR611897	−	−	Hartley (2020)
*Rosellinia merrillii*	HAST89112601	GU300071	−	GQ844781	GQ470229	Hsieh et al. (2010)
*Rosellinia thelena*	CBS 400.61	KF719202	KF719215	−	−	Wu et al. (2025)
*Ruwenzoria pseudoannulata*	MUCL 51394^T^	KY610406	KY610494	KY624286	KX271278	Wendt et al. (2018)
*Sarcoxylon compunctum*	CBS 359.61	KT281903	KT281898	KY624230	KX271255	Senanayake et al. (2015)
*Selenodriella cubensis*	CBS 683.96^T^	KP859053	KP858990	−	−	Hernández-Restrepo et al. (2017)
*Seynesia erumpens*	SMH 1291	−	AF279410	AY641073	−	Bhattacharya et al. (2000)
*Sporidesmina malabarica*	NN43118	−	DQ408580	−	−	Bhattacharya et al. (2000)
*Sporidesmina polypora*	NN47796	OL627678	DQ408569	DQ435087	−	Delgado et al. (2025)
*Stilbohypoxylon elaeidicola*	HAST 94082615	GU322440	−	GQ844827	GQ495933	Hsieh et al. (2010)
*Stilbohypoxylon elaeidis*	MFLUCC 15-0295b	MT496746	MT496756	−	−	Konta et al. (2020)
*Stromatoneurospora phoenix*	BCC82040	MT703666	MT735133	MT742605	MT700438	Becker et al. (2020)
*Thamnomyces dendroideus*	CBS 123578^T^	FN428831	KY610467	KY624232	KY624313	Stadler et al. (2010)
*Theissenia cinerea*	YMJ 90071615^T^	EF026128	−	JX507793	EF025613	Hsieh et al. (2010)
*Thuemenella cubispora*	CBS 119807	JX658531	EF562508	−	−	Stadler et al. (2014)
*Vamsapriya bambusicola*	MFLUCC 11-0477^T^	KM462835	KM462836	KM462834	KM462833	Dai et al. (2014)
*Vamsapriya breviconidiophora*	MFLUCC 14-0436^T^	MF621584	MF621588	−	−	Li et al. (2017)
*Vamsapriya indica*	MFLUCC 12-0544	KM462839	KM462840	KM462841	KM462838	Dai et al. (2014)
*Vamsapriya khunkonensis*	MFLUCC 11-0475^T^	MW240620	MW240549	KM462829	KM462828	Dai et al. (2014)
*Vesiculozygosporium echinosporum*	CPC 35607^T^	MT223869	MT223940	−	−	Crous et al. (2020)
*Virgaria nigra*	CBS 128006	MH864744	MH876180	−	−	Vu et al. (2019)
*Xenoanthostomella chromolaenae*	MFLUCC 17-1484^T^	MN638863	MN638848	MN648729	−	Hyde et al. (2020a)
*Xenoanthostomella cycadis*	CPC17285	KJ869121	KJ869178	−	−	Crous et al. (2014)
*Xylaria allantoidea*	BCC22746	MT703671	MT735141	MT742610	−	Becker et al. (2020)
*Xylaria atrosphaerica*	HAST91111214	GU322459	−	GQ848342	GQ495953	Hsieh et al. (2010)
*Xylaria digitate*	HAST919	GU322456	−	GQ848338	GQ495949	Hsieh et al. (2010)
*Xylaria heliscus*	HAST88113010	GU324742	−	GQ848355	GQ502691	Hsieh et al. (2010)
*Xylaria hypoxylon*	CBS122620^T^	AM993141	KM186301	KM186302	KM186300	Peršoh et al. (2009)
*Xylaria longipes*	CBS 148.73	MH860649	MH872351	KU684280	KU684204	Vu et al. (2019)
*Xylaria polymorpha*	MUCL 49884	KY610408	KY610464	KY624288	KX271280	Wendt et al. (2018)
*Xylaria regalis*	HAST92072001	GU324744	−	GQ848357	GQ502693	Hsieh et al. (2010)
*Zygosporium minus*	HKAS 99625^T^	MF621586	MF621590	−	−	Li et al. (2017)
*Zygosporium oscheoides*	MFLUCC 14-0402^T^	MF621585	MF621589	−	−	Li et al. (2017)

Newly generated sequences are bold.

AFTOL-ID, Assembling the Fungal Tree of Life; ATCC, The American Type Culture Collection; BAFC, Faculty of Exact and Natural Sciences, University of Buenos Aires; BCC, Biotec Culture Collection; BCMX, Baja California Mexico; BISH, Herbarium Pacificum; CBS, Westerdijk Fungal Biodiversity Institute; CPC, Culture Collection of Pedro Crous; DAOM, Canadian National Mycological Herbarium; FMR, Facultad de Medicina, Spain; GBMC, Global Microbiome Conservancy; HAST, Biodiversity Research Museum, Herbarium Academia Sinica; HKAS, Cryptogamic Herbarium of Kunming Institute of Botany; HKU-M, Hong Kong University Herbarium; HKUCC, Hong Kong University Culture Collection; HUEFS, Herbarium of the University of Helsinki; ICMP, International Collection of Microorganisms from Plants; JCM, Japan Collection of Microorganisms; KNU, Kyungpook National University, Korea; MFLU, Mae Fah Luang University Herbarium; MFLUCC, Mae Fah Luang University Culture Collection; MUCL, Agro-food & Environmental Fungal Collection; NBRC, National Institute of Technology and Evaluation; NRRL, Agricultural Research Service Culture Collection; PDD, New Zealand Fungarium; PUFD, Culture Collection at Pondicherry University; PUFNI, University of Pondicherry; SMH, National Museum of Natural History; SQUCC, Sultan Qaboos University Culture Collection; STMA, Deutsche Sammlung von Mikroorganismen und Zellkulturen microbial culture collection; TASM, Tasmanian Fungal Culture Collection; UCD, The U.C. Davis Viticulture and Enology Department Culture Collection; WA, The Western Australian Microbial Culture Collection; WSP, Herbarium at Washington State University; WU, Herbarium of University of Vienna; YMJ, The Japan Collection of Microorganisms.

### 2.3 Phylogenetic analyses

Newly generated sequences were subjected to BLASTn searches against the GenBank database (http://www.ncbi.nlm.nih.gov/) to determine preliminary taxonomic affiliations. Additional sequences were obtained from GenBank based on Basic Local Alignment Search Tool (BLAST) results and previous studies. A concatenated dataset of LSU, ITS, RPB2, and TUB2 sequences was assembled, comprising 202 strains. Leptosillia cordylines (MFLU 20-0056) and Delonicicola siamense (MFLUCC 15-0670) were selected as the outgroup.

Individual gene alignments were generated using the online version of Multiple Alignment using Fast Fourier Transform (MAFFT) v. 7.0362 (Katoh et al., 2019) with default parameters and manually refined in BioEdit 7.1.3 (Hall, 1999) where necessary. Maximum Likelihood (ML) analysis was performed with Randomized Axelerated Maximum Likelihood (RAxML) (Stamatakis and Alachiotis, 2010) implemented in raxmlGUI v.1.5 (Silvestro and Michalak, 2012) using the ML + rapid bootstrap setting and the general time-reversible (GTR) + I + G model of nucleotide substitution with 1,000 rapid bootstrap replicates. For Bayesian inference (BI), the optimal substitution model (GTR+G+I) was determined using MrModeltest software v. 2.2 (Nylander, 2004). BI analysis was conducted in MrBayes v. 3.2.6 (Ronquist et al., 2012) with four simultaneous Markov chain Monte Carlo chains from random trees over 3 million generations (standard deviation of split frequencies < 0.01), sampling every 500^th^ generation. The distribution of log-likelihood scores was detected to check whether sampling was in the stationary phase, and Tracer v1.5 was used to check if further runs were required to reach convergence (Rambaut and Drummond, 2007). The first 20% of sampled trees were discarded as burn-in before calculating a consensus tree and posterior probabilities. Resultant phylogenetic trees were visualized using FigTree v.1.4 (Rambaut, 2012).

### 2.4 De novo genome assembly and genome sequencing of Dianjunus rex

Whole-genome and transcriptome sequencing were performed on three stromata of Dianjunus rex collected from the wild. Internal tissue was excised from each stroma. High-quality genomic DNA was extracted from each sample using the cetyltrimethylammonium bromide (CTAB)method (Porebski et al., 1997). Total RNA was extracted with TRIzol^®^ Reagent (Invitrogen) extraction kit, followed by purification using the Plant RNA Purification Reagent (Invitrogen) purification kit. Short DNA and RNA reads were generated for each sample on the Illumina HiSeq 4000 platform (San Diego, CA, USA). These reads were then trimmed and filtered using fastp (v0.23.2) (Chen et al., 2018) with parameters ‘-f 10 and -F 10’ to remove adapter sequences and low-quality reads. For paired-end DNA sequencing data, afterthe clean data converted were into a FASTA file, 10,000 reads were randomly selected from each file and aligned against the NCBI Nucleotide (NT) database, indicating no significant contamination. Finally, in total, we obtained 49 Gb of clean data for DNA and 51 Gb for RNA, respectively. For genome assembly, the filtered DNA reads from each sample were assembled independently using SPAdes (v4.2.0) (Bankevich et al., 2012). Genome completeness was assessed by Benchmarking Universal Single-Copy Orthologs (BUSCO, v5.2.2) (Simão et al., 2015) with the ascomycota_odb10 orthologous gene set.

### 2.5 Annotation of repetitive sequences and protein-coding genes

RepeatModeler (v2.0.2) (Flynn et al., 2020) was used to build the de novo repeat libraries and then sequences from the assembly were aligned to the de novo repeat libraries and known repeat databases to identify and mask repetitive elements, using RepeatMasker (v4.1.2) (Chen, 2004). Protein-coding genes in each genome were predicted using the Genome-wide Electronic Tool for Annotation (GETA, v2.6.1) pipeline (https://github.com/chenlianfu/geta), integrating evidence from homology-based, ab initio, and RNA-seq-based prediction approaches. For homology-based prediction, protein-coding sequences of Biscogniauxia marginata, Annulohypoxylon maeteangense, Daldinia caldariorum, Hypoxylon argillaceum, Astrocystis sublimbata, Durotheca rogersii, Kretzschmaria deusta, Nemania abortiva, Poronia punctata, and Xylaria curta were downloaded from the Joint Genome Institute (JGI) (Franco et al., 2022) and were used for gene prediction using GeneWise (v2.4.1) (Birney et al., 2004). The ab initio prediction was conducted using AUGUSTUS (v3.4.0) (Stanke et al., 2006). RNA-seq reads were aligned to the genome assembly using hierarchical indexing for spliced alignment of transcripts 2 (HISAT2, v2.1.0) (Kim et al., 2015), and protein-coding regions were predicted using TransDecoder (v5.5.0) (https://github.com/TransDecoder/TransDecoder). The predictions from the three methods were then integrated and filtered against the Pfam database.

### 2.6 Phylogenetic tree construction and divergence time estimation

Protein sequences of 63 species in Pezizomycotina were downloaded from JGI Mycocosm database and combined with sequences from three samples generated in this study, resulting in 66 samples for phylogenetic analysis. Single-copy orthologous proteins conserved across all 66 samples were retrieved from the results of BUSCO assessment. A total of 302 single-copy orthologs were then aligned using MAFFT (v7.407) (Katoh et al., 2002) with default parameters, and the alignments were concatenated sequentially to construct a super matrix. The phylogenetic tree was constructed from this super matrix using IQ-TREE 3 (v3.0.1) (Wong et al., 2026), with Aspergillus niger and A. tetrazonus serving as the outgroup. Branch supports were assessed using a bootstrap approach with 1,000 replicates. The species tree was visualized using the web application Tree Visualization By One Table (tvBOT) (Xie et al., 2023).

To further cross-verify the topological stability across different ortholog selection workflows, an alternative dataset was constructed. Orthologous groups across the same 66 samples were clustered using OrthoFinder (v3.1.3) with the DIAMOND alignment mode (-S diamond) and MAFFT for sequence alignment (-M msa -A mafft), yielding 345 single-copy orthologous gene sets. The amino acid sequences of each orthogroup were aligned using MAFFT (v7.407) with automatic parameter selection (--auto). To eliminate poorly aligned or highly divergent regions, the alignments were systematically trimmed using trimAl (v1.4.rev15) with a gap threshold of 0.3 (-gt 0.3), ensuring that sites with more than 70% gaps were removed. Individual maximum-likelihood gene trees were then inferred from the trimmed alignments using IQ-TREE 3 (v3.0.1) under the best-fit evolutionary models automatically selected using ModelFinder Plus (MFP) with 1,000 ultrafast bootstrap replicates, utilizing the same outgroup (*Aspergillus niger* and *A. tetrazonus*). Finally, these 345 gene trees were used as input for Accurate Species TRee ALgorithm (ASTRAL, v5.7.1) to reconstruct the alternative coalescent-based species tree.

The MCMCtree module of the Phylogenetic Analysis by Maximum Likelihood (PAML)package (v4.9i) (Yang, 2007) was used to estimate each speciation time for these 66 Pezizomycotina samples. The phylogenetic tree constructed by IQ-TREE 3 served as a guide tree, with the divergence times from TimeTree (Kumar et al., 2017) used as calibration points: Aspergillus tetrazonus and Pyricularia oryzae (233.8~367.0 million years ago [MYA]), Pseudomassariella vexata and Microdochium trichocladiopsis (143.3~164.1 MYA), Microdochium bolleyi and Hypoxylon fuscum (103.4~318.2 MYA), and Colletorichum cereale and Verticillium longisporum (85.0~139.4 MYA).

### 2.7 Identification and analysis of carbohydrate-active enzymes

Carbohydrate-active enzymes (CAZymes) profiles were identified in the proteomes of all 66 Pezizomycotina samples. BLAST and hidden Markov model (HMM) searches were performed simultaneously using the proteomes as queries to detect putative CAZymes. For the BLAST searches, query protein sequences were used as inputs in the BLASTp tool against the database for automated CAZyme annotation (dbCAN, v11) (Yin et al., 2012). The resulting hits were filtered by e-value (1e-5) and identity threshold (20%). For the HMM search, the hmmscan program in the HMMER package was used to match the family-specific HMM profiles of CAZymes within dbCAN database (v11). The candidate genes obtained from both the BLAST and HMM searches were merged and only the overlapping hits were retained. The raw sequencing data have been deposited in the National Center for Biotechnology Information (NCBI) under the BioProject accession number of PRJNA1265937. All genome assemblies and gene annotation results have been archived in the figshare database and can be accessed at https://doi.org/10.6084/m9.figshare.29153135.v2. The multilocus alignment of this study can be accessed via https://doi.org/10.6084/m9.figshare.32611812.

## 3 Results

### 3.1 Phylogeny

All individual trees generated under different criteria and from single-gene datasets were essentially similar in topology and not significantly different from the tree generated from the concatenated dataset (not discussed herein). Maximum likelihood analysis of new strains generated in this study and other related taxa in this study with 1,000 bootstrap replicates yielded the best ML tree (**Figure 1**) with the likelihood value of ln: –6728.286175 and the following model parameters: alpha: 0.191444; Π(A): 0.244188, Π(C): 0.230054, Π(G): 0.301322, and Π(T): 0.224436. The substitution rates are as follows: AC: 0.763664, AG: 2.779076, AT: 1.349374, CG: 0.639806, CT: 6.128235, GT: 1.0. The Bayesian analysis resulted in 10,000 trees after 5,000,000 generations, and the first 20% of the trees were discarded. The remaining trees were used for calculating posterior probabilities in the majority-rule consensus tree. Maximum likelihood bootstrap values ≥50% and Bayesian inference (BI) ≥0.9 are given at each node. Tree topologies of the ML and Bayesian analyses were similar to each other and there were no significant differences. Both topologies showed 15 well-supported major clades representing families in *Xylariales*. These new fungal collections grouped as a sister clade to *Graphostromataceae* with ML/BI = 100%/1.00 support.

**Figure 1:**
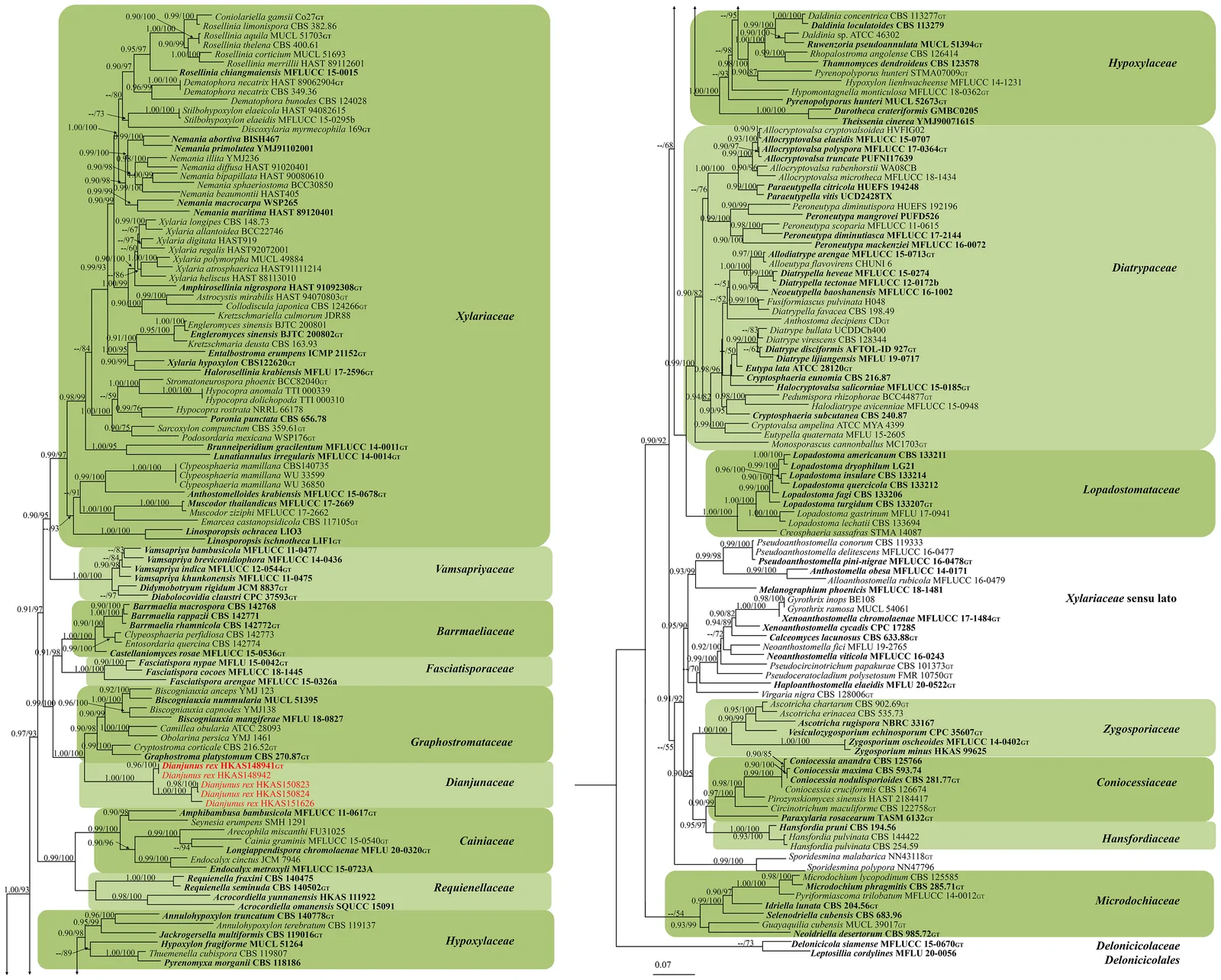
Phylogenetic tree inferred from maximum likelihood analysis of the combined ITS, LSU, *TUB2*, and *RPB2* sequence alignment. Nodes are labeled with maximum likelihood bootstrap support values ≥50%, and Bayesian posterior probabilities ≥0.90. The tree was rooted with Leptosillia cordylines (MFLU 20-0056) and Delonicicola siamense (MFLUCC 15-0670). Ex-type cultures are denoted in bold, and sequences newly generated in this study are highlighted in red. Generic types are indicated by “^GT^”.

Phylogenomic analysis confirmed that *Dianjunus rex* represents a distinct lineage within *Dianjunaceae* (**Figure 2**). Comparative CAZyme annotation revealed an exceptionally compact and highly conserved enzyme repository across the three sequenced *D. rex* strains (HKAS151626, HKAS148941, and HKAS150823), encoding 220, 224, and 219 total CAZyme genes, respectively.

**Figure 2:**
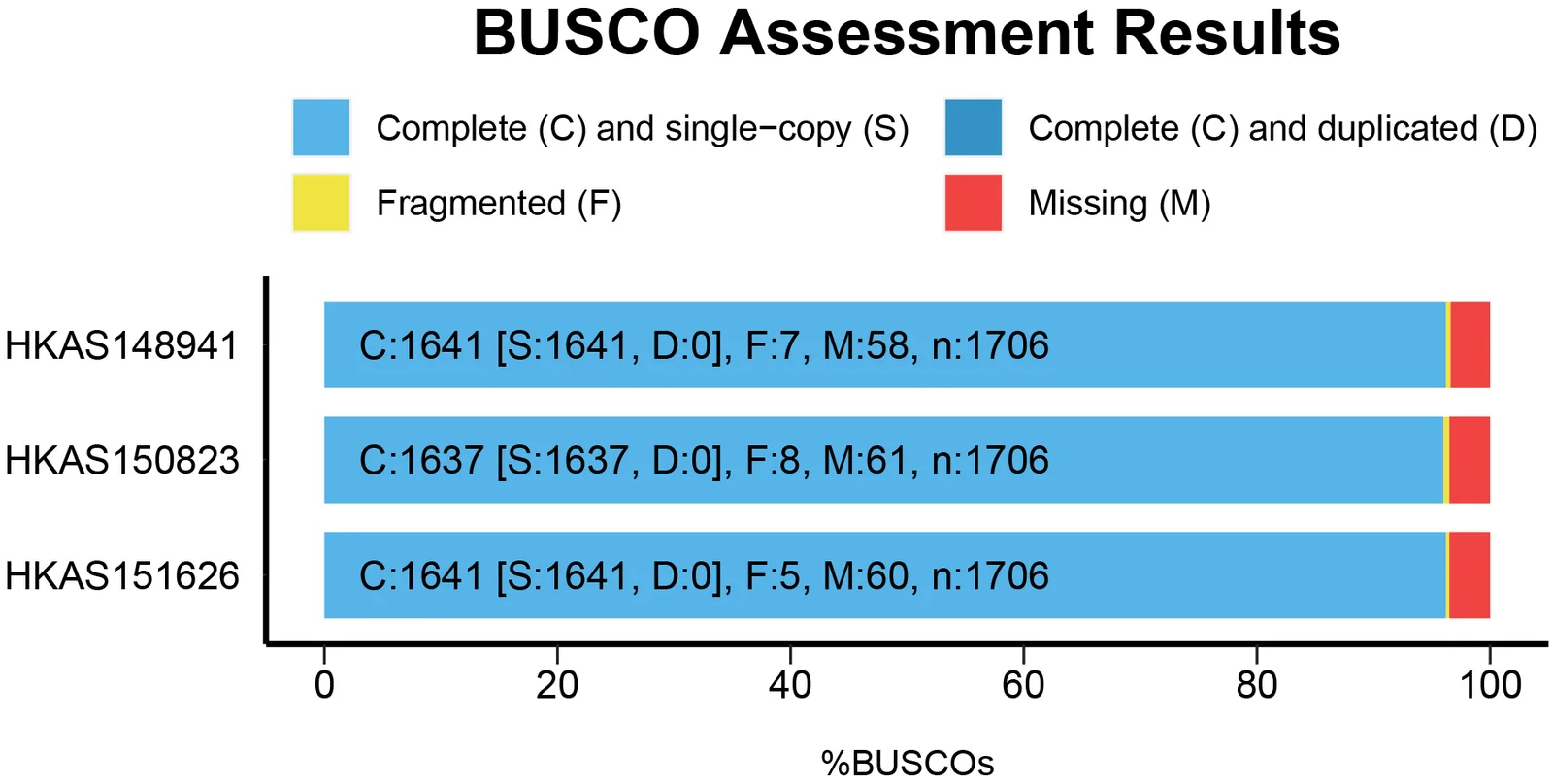
BUSCO assessments of the genome assembly and proteins of *Dianjunus* rex.

Across the broad phylogenomic tree of *Sordariomycetes* and related *Xylariales* (**Figure 2**), *D. rex* exhibits the most severe species-wide CAZyme contraction observed. Compared to typical saprobic and wood-decaying lineages such as *Xylaria* (535–635 genes), *Daldinia* (458–496 genes), and *Hypoxylon* (423–577 genes), the total CAZyme burden of *D. rex* is reduced by more than half. Specifically, Glycoside Hydrolases (GHs: 100–103) and Carbohydrate Esterases (CEs: 9–11) are markedly diminished, and Pectin Lyases (PLs) are virtually depleted (PL = 0-1; completely absent in strain HKAS148941) compared to 7–19 PL genes in closely related *Xylariales* lineages (**Figure 2**). Conversely, Glycosyltransferases (GTs: 57–58) represent a disproportionately high fraction (~25.9%–26.0%) of its overall enzymatic repertoire.

### 3.2 Phylogenomic tree construction and divergence time estimation

The genome assembly sizes of samples HKAS148941, HKAS150823, and HKAS151626 are 31.09 Mb, 32.01 Mb, and 30.48 Mb with contig N50 values of 47.63 kb, 42.61 kb, and 76.62 kb respectively (**Table 3**). Genome completeness assessment revealed that at least 96.19%, 95.96%, and 96.19% of single-copy BUSCO genes were identified in the assembly (**Figure 3**, **Table 3**).

**Table 3:** Genome assembly statistics of *Dianjunus* rex.

Sample	Contig N50 (kb)	Contig Num	Total length (Mb)	Protein-Coding genes
HKAS148941	47.63	1471	31.19	7793
HKAS150823	42.61	1437	32.01	6753
HKAS151626	76.62	889	30.48	6779

**Figure 3:**
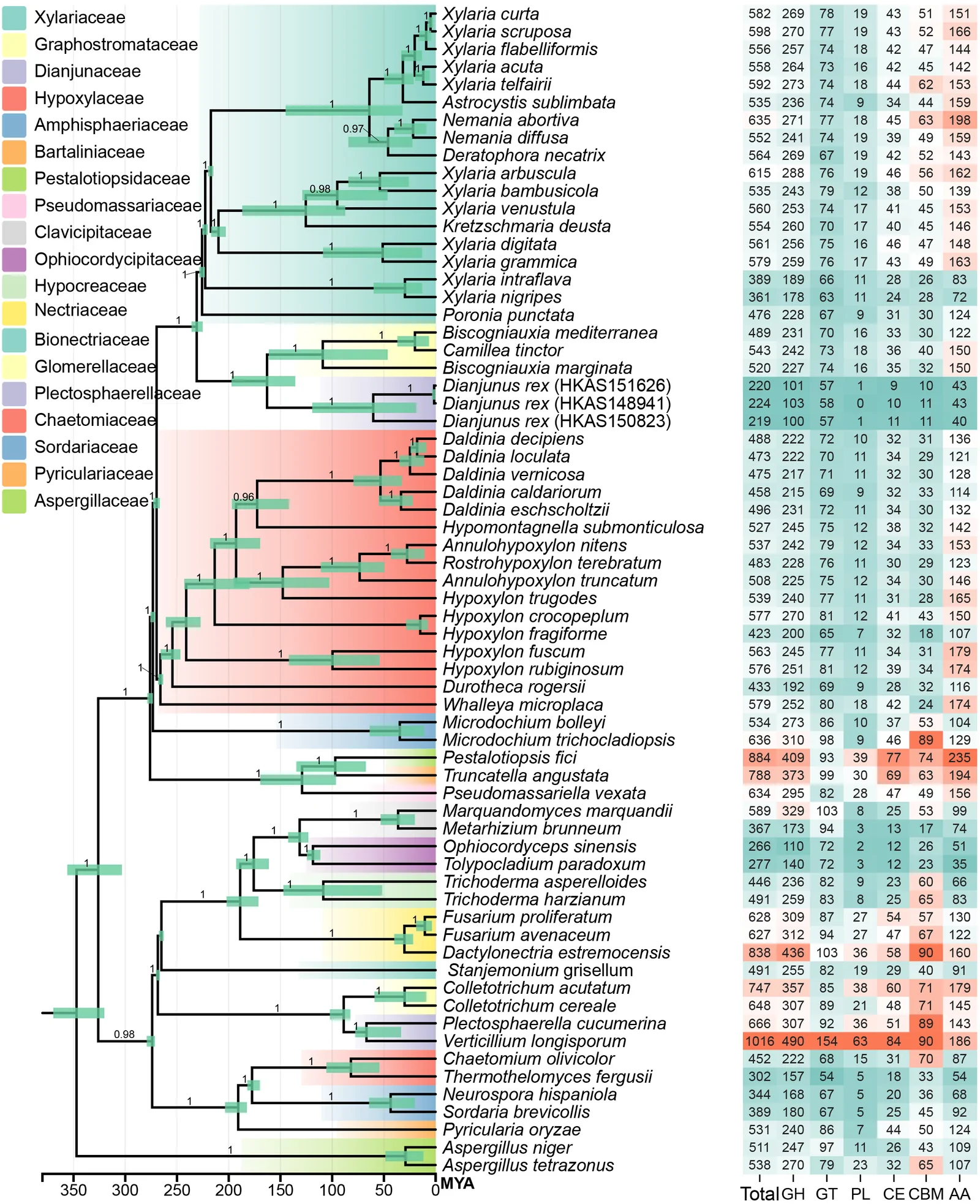
Phylogenomic tree inferred from a genome-scale dataset comprising 66 Pezizomycotina species with Aspergillus niger and A. tetrazonus asoutgroup taxa. Devonian (420–360 Ma), Carboniferous (360–300 Ma), Permian (300–250 Ma), Triassic (250–200 Ma), Jurassic (200–145 Ma),Cretaceous (145–65 Ma), Paleogene (65–23 Ma), Neogene (23–2.6 Ma).

### 3.3 Taxonomy

*Dianjunaceae* F.Q. Yu, Pérez-Moreno & Senan., fam. nov.

Index Fungorum number: IF904757

Etymology: Based on the type genus Dianjunus.

Diagnosis: Dianjunaceae is distinguished from other families in Xylariales by having large stromata with persistent powdery-fibrous surface, unique S-shaped germ slit in ascospores, the inverted-trapezoid, amyloid apical ring in Melzer’s reagent, and nodulisporium-like anamorph with periconiella-like branching patterns produce distantly-packed branchlets bearing cluster of cylindrical, conidiogenous cells.

Type genus: Dianjunus F.Q. Yu, Pérez-Moreno & Senan.

Teleomorph: Stromata upright, fleshy, variable in size, shape ranging from cylindrical, fusiform, turbinate to palm-shaped, terete to flattened, simple to branched from the base, sometimes arising from long rooting stipes, with flattened sterile apices. Outer crust coriaceous, immersed part in soil black, aerial part fertile, surface covered with a long persistent, hairy, whitish yellow fibrous substance, whitish in young stromata, turning progressively dark yellow at maturity, ostiole openings appear as black dots. Immersed part sterile, consisting of stipes, cylindrical, black, smooth. Internal tissues solid, homogeneous to fibrous, spongy, off-white to yellow, with a slightly darker core in aged specimens. Perithecia aggregated, immersed in stromata, subglobose, coriaceous, papillate, ostiolate. Ostioles raised-discoid, black, with a cylindrical papilla at center. Paraphyses sparse, hypha-like, narrowing toward the ends, hyaline, septate, slightly constricted at the septa, unbranched. Asci (6–)8-spored, unitunicate, cylindrical, long-stipitate, total length, with apical apparatus, appearing bilobed, bluing in Melzer’s reagent, reddish brown in Lugol’s solution, turning blue in Lugol’s solution after treatment with 3% (w/v) KOH. Ascospores overlapping uniseriate in the ascus, globose to oval or fusiform with acute ends, hyaline when young, becoming brown at maturity, with single, central guttule or with a large central and two small polar guttules, smooth, thick-walled, with a conspicuous, S-shaped germ slit one-half to four-fifths spore length on the flattened side. Anamorph: nodulisporium-like anamorph with periconiella-like branching patterns.

Notes: The multilocus phylogeny of ITS, LSU, TUB2, and RPB2 sequences showed that our new fungal collections clustered together forming a well-supported, distinct sister clade to Graphostromataceae in Xylariales. However, morphological characteristics of our collections are significantly different from those of species in this family. Further, only few genera in Graphostromataceae, such as Camillea and Biscogniauxia, produce upright stromata. The phylogenomic analyses also confirmed the placement of these collections as sister to Graphostromataceae in Xylariales. Therefore, we introduce this unidentified clade as a new family Dianjunaceae.

*Dianjunus* F.Q. Yu, Pérez-Moreno & Senan., gen. nov.

Index Fungorum number: IF904733

Etymology: The generic name is derived from the “Dian Kingdom”, a historical society situated in the region of present-day Yunnan Province in southwestern China where the samples were collected. The epithet is combined with the Chinese word “jūn”, meaning ‘mushroom’ or ‘fungus’.

Type species: Dianjunus rex F.Q. Yu, Pérez-Moreno & Senan.

Arise from roots of dying host plants. Teleomorph: Stromata upright, fleshy, variable size and shape, ranging from cylindrical, fusiform, turbinate to palm-shaped, terete to flattened, simple to branched from the base, or at the top, arising from long rooting stipes, with flattened sterile apices. Outer crust coriaceous, black, ostiole openings appear as black dots. Aerial part fertile, surface covered with a long persistent, powdery, whitish yellow fibrous substance, whitish in young stromata, turning progressively dark yellow at maturity. Immersed part sterile, consisting of stipes, cylindrical, black, no powdery substance, smooth. Interior tissues solid, homogeneous to fibrous, spongy, off-white to yellow, with a slightly darker core in aged specimens, absence of discolouration with 5% (w/v) KOH. Ascomata perithecial, aggregated, scattered, immersed in stromata, subglobose, coriaceous, papillate, ostiolate. Ostioles raised-discoid, black, with a central, cylindrical papilla. Peridium dark brown, turning subhyaline inwardly, containing cells of textura porrecta mixed with textura angularis, evenly thickened. Paraphyses sparse, hypha-like, narrowing toward the ends, hyaline, septate, slightly constricted at the septa, unbranched, longer than asci, embedded in mucilaginous matrix. Asci (6–)8-spored, unitunicate, cylindrical, long-stipitate, total length, with apical apparatus, appearing bilobed, amyloid, cuboid in Melzer’s reagent, reddish brown in Lugol’s solution, turning blue in Lugol’s solution after treatment with 3% (w/v) KOH, inverted trapezoid. Ascospores overlapping uniseriate in the ascus, globose to oval or fusiform with acute ends, hyaline when young, becoming brown at maturity, with single, central guttule or with a large central and two small polar guttules, smooth, thick-walled, with a conspicuous, S-shaped germ slit one-half to four-fifths spore length on the flattened side. Anamorph: nodulisporium-like with periconiella-like branching patterns.

Notes: The phylogenetic analyses of combined ITS, LSU, TUB2, and RPB2 loci and phylogenomic analyses showed that these five collections (HKAS150824, HKAS151626, HKAS150823, HKAS148941, HKAS148942) clustered forming a well-supported clade (**Figure 1**). Morphological characters of collections in this clade are significantly different from those of sister clades. Therefore, we establish this clade as Dianjunus gen. nov.

*Dianjunus rex* F.Q. Yu, Pérez-Moreno & Senan., sp. nov. **Figures 4**–**6**

**Figure 4:**
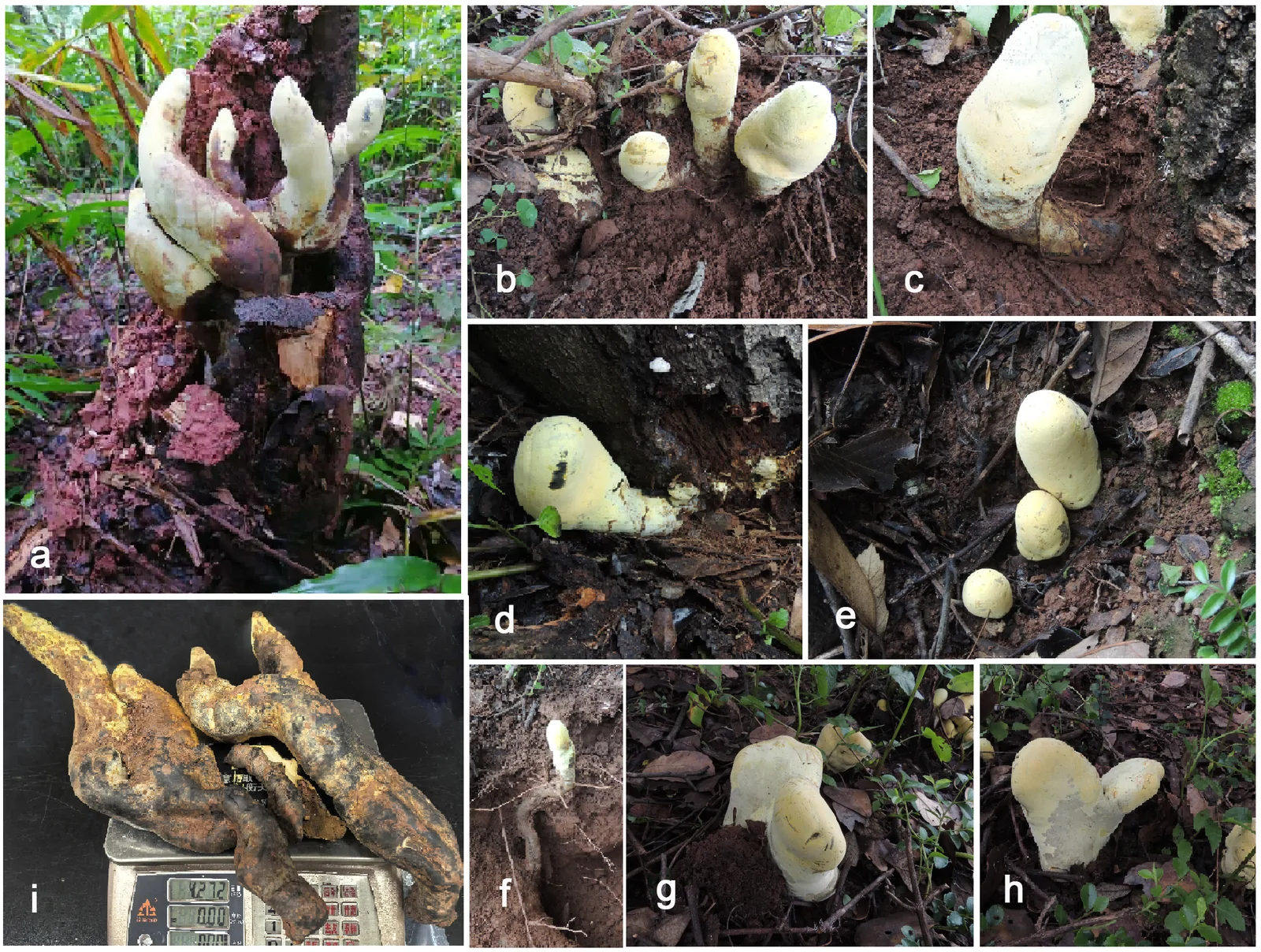
Stromata of *Dianjunus rex* in the field. **(a, c, d, f)** Stromata attached to host roots. **(b, e, g, h)** Stromata around host plant. **(a)** Collectors. **(i)** Total weight of largest bilobedstromata.

**Figure 5:**
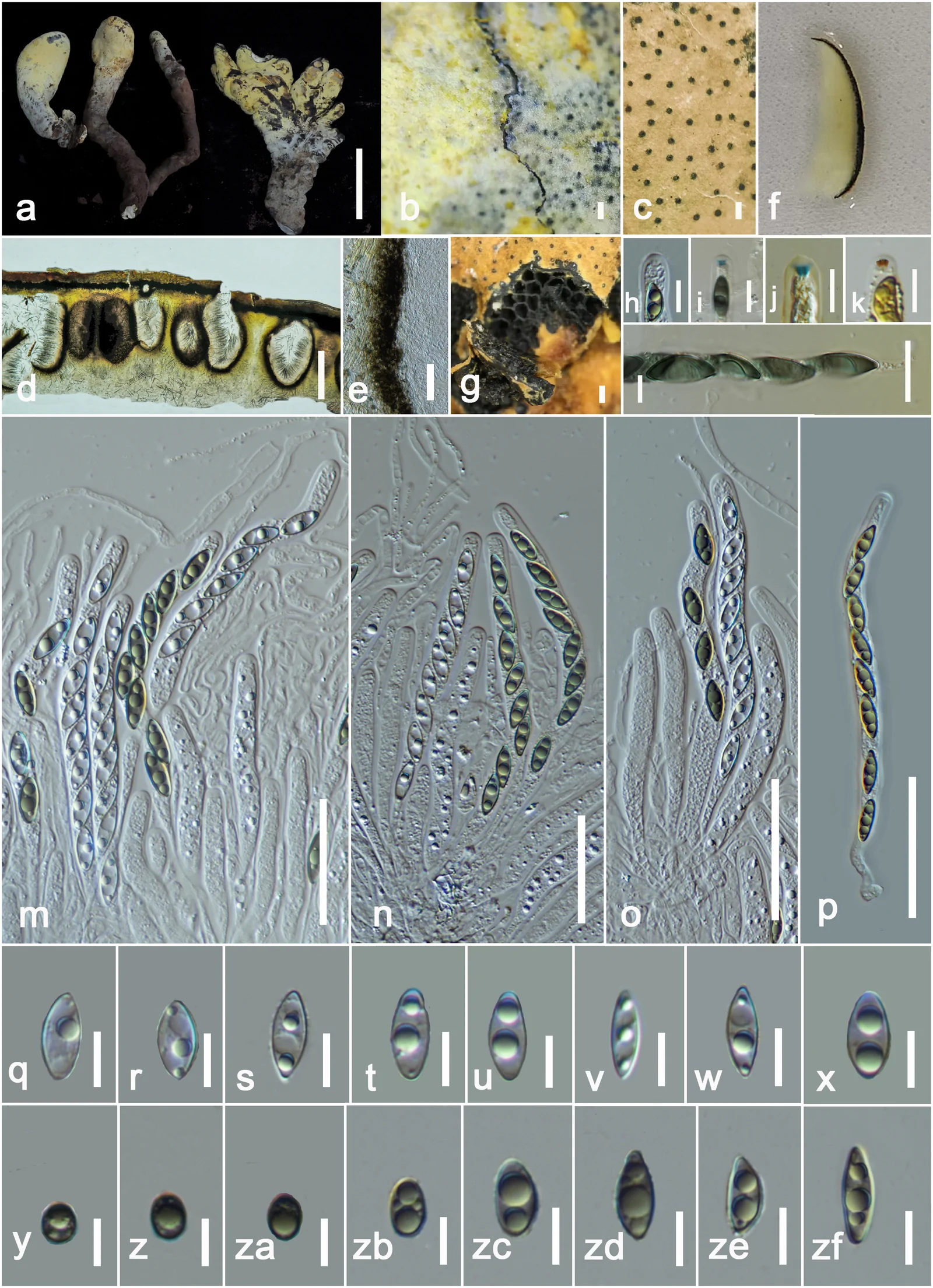
*Dianjunus rex* (HKAS148941, holotype). **(a)** Stromata. **(b)** Yellow powdery layer on outer crust covering ostioles. **(c)** Ostiole opening on stromatic surface. **(d)** Vertical cross section of ascoma. **(e)** Peridium. **(f)** Stromatic tissues in 5% (w/v) KOH. **(g)** Horizontal section through the stromata. **(h)** Apical ring in water. **(i)** Apical ring in Melzer’s reagent. **(j)** Apical ring in Lugol’s reagent after pretreatment with 3% (w/v) KOH. **(k)** Apical ring in Lugol’s reagent. **(l)** S-shaped germ-slits in ascospores in Melzer’s reagent. **(m-p)** Asci in water. **(q-zf)** Ascospores in water. Scale bars: a = 15 cm, b, c, e = 100 µm, d = 750 µm, g = 250 µm, l = 20 µm, m-p = 50 µm, h-k, q-zf = 10 µm.

**Figure 6:**
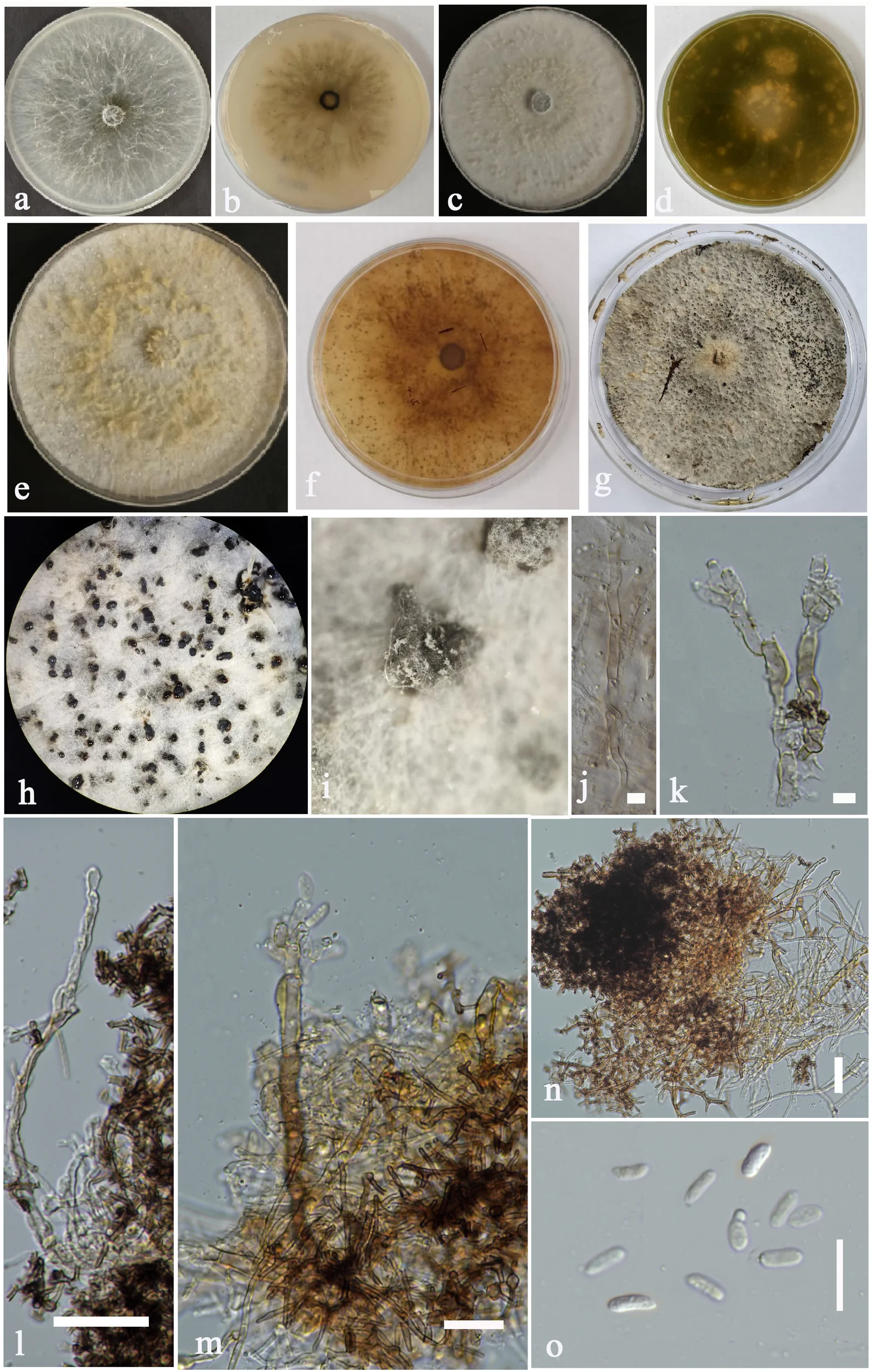
Cultures and anamorph of *Dianjunus rex* (GBWSCC202508860). **(a, c, e)** Surface view of culture on MEA, OMA, and PDA respectively after a week. **(b, d, f)** Reverse view of culture on MEA, OMA, and PDA after a week. **(g, h)** 90-day-old sporulated culture on PDA. **(i)** Close-up view of spore-bearing structures. **(j)** Mycelia. **(k-m)** Conidiophores, conidiogenous cells with attached conidia. **(n)** Specialized mycelial cells from which conidiophores are derived. **(o)** Conidia. Scale bars: **l-n** = 20 µm, **j,k,o** = 10 µm.

Index Fungorum number: IF904732

Etymology: The species epithet rex (Latin = king) refers to the large size and majestic appearance of the stromata.

Type: China, Yunnan Province, Kunming City, Luquan Yi and Miao Autonomous County, Tuanjie Town, Damaidi, associated with roots of Quercus variabilis Blume (Fagaceae), 5 August 2024, Jing Song, (HKAS148941, holotype), ex-type culture GBWSCC202508860, HKAS151626 (dried culture); ibid. 6 September 2024, Senanayake IC (HKAS148942, Paratype).

From roots of dying host plants and arise from rhizosphere soil. Teleomorph: Stromata upright, slender to plump fleshy, 20–50 cm total height, with fertile parts 10–20 cm high, 7–10(–15) cm diam., 250–600 g weight, variable size and shape, ranging from cylindrical, fusiform, turbinate to palm-shaped, terete to flattened, simple to branched from the base, or at the top, arising from long rooting stipes, with flattened sterile apices. Outer crust 200–300 μm thick, coriaceous, black, ostiole openings appear as black dots, ostiolar papillae are distributed in this layer. Aerial part fertile, surface covered with a long persistent, powdery, whitish yellow fibrous substance, whitish in young stromata, turning progressively dark yellow at maturity. Immersed part 20–30 cm high, 3–6 cm wide, sterile, consisting of stipes, cylindrical, black, no powdery substance, smooth. Interior tissues solid, homogeneous to fibrous, spongy, off-white to yellow, with a slightly darker core in aged specimens, absence of discoloration with 5% (w/v) KOH. Ascomata 400–750 μm diam., 1200–1500 μm high, perithecial, aggregated, scattered, immersed in stromata, subglobose, coriaceous, centrally papillate, ostiolate. Ostioles 150–280 μm diam., raised-discoid, black, with a cylindrical papilla at center. Paraphyses 4–7 μm broad (x̄ = 5 μm), sparse, hyphal, thin-walled, narrowing toward the ends, hyaline, septate, slightly constricted at the septa, unbranched, longer than asci, embedded in mucilaginous matrix. Peridium 70–100 µm (x̄ = 80 μm), thick, dark brown, turning subhyaline inwardly, containing cells of textura porrecta mixed with textura angularis, evenly ticked. Asci (6–)8-spored, unitunicate, cylindrical, long-stipitate, 115–160 × 8–10 µm (x̄ = 2.5 × 3 μm) total length, the spore-bearing parts 90–100 × 8–10 µm, with bilobed, apical apparatus, 2–3 µm high, 3–5 µm wide (x̄ = 3 × 4 μm) in water; cuboid, amyloid, 2–3 µm high, 2.8–3.2 µm wide (x̄ = 2.5 × 3 μm), in Melzer’s reagent; inverted trapezoid, reddish brown in Lugol’s solution, 2.5–3 µm high, 3–4.5 µm wide (x̄ = 3 × 4 μm), turning blue in Lugol’s solution after treatment with 3% (w/v) KOH. Ascospores 12–17 × 5–7 µm, overlapping uniseriate in the ascus, globose to oval or fusiform with acute ends, hyaline when young, becoming brown at maturity, with single, central guttule or with a large central and two small polar guttules, smooth, thick-walled, with a conspicuous, S-shaped germ slit one-half to four-fifths spore length on the flattened side. **Anamorph**: nodulisporium-like anamorph with periconiella-like branching patterns. Mycelium consisting of septate, branched, smooth-walled hyphae, hyaline to pale brown. Conidiophores 17–25 µm length, 6–8 µm width (x̄ = 20 × 7 µm, n = 10), macronematous, mononematous, erect, septate, smooth, pale to dark brown, arising singly from hyphae; branching profuse and arborescent (periconiella-like), with primary and secondary branches producing numerous short terminal branchlets. Conidiogenous cells 7–10 µm length, 3–6 µm width (x̄ = 9 × 5 µm, n = 20), terminal, polyblastic, sympodially proliferating (nodulisporium-like), denticulate; conidiogenous loci minute, with small truncate scars. Conidia 6–9 µm length, 3–5 µm width (x̄ = 8 × 4 µm, n = 25), produced singly on denticles, ellipsoid to ovoid, hyaline, smooth, unicellular.

Culture characteristics: Colonies after 7 days of incubation at 20 °C in dark; on PDA reaching 5 cm diam., circular, smooth margin, flat, bearing abundant aerial mycelium, initially white, turning to yellowish-white with time, lack of pigment production, heavily sporulated with black, globose conidiomata; on MEA reaching 4.5 cm, circular, thin, arachnoid, with abundant, initially white, later turning to blackish brown, radiating, dendritic hyphae; center compact, slightly olivaceous to grayish; margin even to diffuse, reverse view blackish, radiating, dendritic hyphae start from the center and spread toward the margin, lack of pigment production, fairly sporulated with black, globose conidiomata; on OMA reaching 5 cm, circular, white, dense, cottony to floccose, with abundant aerial mycelium, center compact and grayish, surface faintly radially striate with weak zonation, margin entire to diffuse, reverse view with circular, dark olivaceous to olive-brown pigmentation; central region buff to cream, sporulation did not occur.

Additional material examined: China, Yunnan Province, Xishuangbanna Dai Autonomous Prefecture, Mengla County, Mengla Town, attached to trunk and root of decaying unidentified tree, 14 July 2025, Xiao Aka, (HKAS150824); ibid., Chuxiong Yi Autonomous Prefecture, Lufeng City, Yipinglang Town, from roots of Quercus sp., 10 July 2025, Xie Tao, (HKAS150823).

Notes: In the combined multilocus phylogeny analysis of ITS, LSU, TUB2, and RPB2, the collections of Dianjunus species clustered together forming a distinct clade with support value of ML/BI = 100%/1.00 (**Figure 1**). The bootstrap value in phylogenomic analyses was also >90% (**Figure 2**). All the collections of Dianjunus rex are morphologically similar, and stromata of tropical collections are larger than those of subtropical collections (**Figure 4**). The young stromata mostly have bulb-like apex and they form finger-like branches when mature. The fresh stromata are very fleshy and dehydrate rapidly after being detached. There is no strong smell or taste from stromata. Therefore, based on morphological characters and phylogeny, these collections are introduced as a new species, Dianjunus rex. This anamorph described above is derived from pure cultures obtained from single-ascospore isolation. Both cultures obtained from single-spore isolation and conidia suspension of sporulated culture were used for molecular sequence comparison. The sequences of the ITS locus of both cultures are identical to each other.

Our collections morphologically resemble the genus *Squamotubera* P. Henn. Hennings (1903) erected the genus *Squamotubera* for the single species, *S. le-ratii* P. Henn., which was collected from Noumea, New Caledonia (island between Australia and New Zealand) from soil. Lloyd (1917) noted that *Squamotubera le-ratii* is similar to *Sarcoxylon compunctum* (Jungh.) Cooke except for its horizontal or upright stromatal development from bark of living plants. Therefore, it was synonymized as *Sarcoxylon le-ratii* (P. Henn.) Lloyd. Rogers (1981) also argued that *Squamotubera* might be congeneric with *Sarcoxylon.* However, considering the upright stromatal development of *Squamotubera* from subterranean sclerotia, Rogers (1981) conserved *Squamotubera* as a distinct genus and mentioned the requirement of further studies to clarify it. The genus *Squamotubera* is only known from its short Latin protologue by Hennings (1903) with no illustrations. Rogers (1981) noted that the holotype specimen of *Squamotubera le-ratii* is not available in specimen repositories (Farlow Herbarium, FH).

In the original description, Hennings (1903) described the stromata of *Squamotubera le-ratii* as “*tuberiform, round depressed with squamose external surface*”. Rogers (1981) amended the description and mentioned, “*the suspicion that stromata arise from subterranean sclerotia*” and “*the exterior is convoluted with smaller wrinkles overall*”. However, our collections do not fit any of these two characteristics. It is neither “*tuberiform”* nor “*squamose or with wrinkles overall*”. Apart from these diagnostic characteristics, there are other important taxonomic traits that do not fit with the description provided by Hennings (1903) or Rogers (1981) such as “*interior pallid subcarnose*” or “*tan, solid with irregular lacunae*”, while in our fungus, the inner context was “*spongy, off-white to mostly yellow*”.

Rogers (1981) described “*Capitate apices quickly become soft with gelatinous areas beneath the perithecial layer*” as a characteristic never recorded in our specimens, whose context always remains fibrous, spongy. Additionally, Rogers (1981) described “Parasite sur les vielles souches de *Casuarina*” [sic] in French, which means “*Parasite on the old stumps of Casuarina* sp.”, a host plant not recorded for our fungus. Germ-slits in ascospores were not reported from *S. le-ratii* by Hennings (1903) and Rogers (1981) while our collections have S-shaped germ-slits. The stromata of *Squamotubera* are 7 cm high and 5 cm wide, while stromata in our collections are 20–50 cm high and 7–10 (-15) cm diam. Therefore, our collections are significantly different from *Squamotubera le-ratii* (**Table 4**).

**Table 4:** Morphological character comparison of collections of *Squamotubera* and *Dianjunus*.

Characters	Collections described in		
	Hennings (1903)	Rogers (1981)	This study
Stromata			
Shape	tuberiform structure that externally recalls young sclerotia of Lentinus tuber-regium. Rounded-depressed	Upright	upright, ranging from cylindrical, fusiform, turbinate to palm-shaped
Size	7 cm long, 5 cm wide, 15 mm thick	up to 5 cm high and 2 cm broad at the somewhat capitate apex.	20–50 cm total height, 7–10 (-15) cm diam.
Outer part			
Color	ash-gray	yellow to olivaceous with brown tones	Black with fertile part whitish when young, turning progressively to light yellow and dark yellow at maturity, with black stipe surfaces.
Texture	multi-layered membranes which can be peeled off in flakes, often scaly-flaky membranes, externally clothed with membranous, sub-papery, scaly coverings.	pulverulent, convoluted with smaller wrinkles overall.	The aerial fertile structures exhibit a persistent powdery-fibrous surface, coriaceous below, while the sterile regions of the cylindrical stipes are smooth.
Inner part			
Color	Pale leather-yellow	Tan	off-white to yellow
Texture	sub-fleshy	Solid with irregular lacunae as a result of convolutions and infolding	solid, homogeneous to fibrous, spongy
Ascomata			
Shape	Ovoid or ellipsoid	Not described.	subglobose
Size	0.8-1 × 0.5-0.8 mm	0.5 mm diam.	0.40–0.75 mm diam., 1.2–1.5 mm high,
Apices	rounded, blunt apices partly protrude in an almost hemispherical manner, slightly prominent, sub-hemispherical.	Not available	immersed in stromata, subglobose, coriaceous, papillate, ostiolate.
Ostioles	sub-hemispherical, punctiform, black	Not available	raised-discoid, black, with a cylindrical papilla at center
Additional characters	Not available	The stromata quickly become soft with gelatinous areas beneath the perithecial layer during wetting. Rather soft, in a layer on the capitate parts of the stromata	Not available
Microscopy of outer part	The outer gray membranes consist of interwoven, colorless, variably thick, often ribbon-like sterile hyphae, and thinner, septate, richly branched hyphae.	Not available	Mycelium consisting of septate, branched, smooth-walled hyphae, hyaline to pale brown.
Microscopy of fertile hyphae	Fertile hyphae septate, branched	Not described	Conidiophores 17–25 µm length, 6–8 µm width, macronematous, mononematous, erect, septate, smooth, pale to dark brown, arising singly from hyphae; branching profuse and arborescent (periconiella-like), with primary and secondary branches producing numerous short terminal branchlets.
**Asci**	cylindrical, 8-spored, uniseriately arranged.	Most asci are immature and agglutinated; measurements were not possible. The ascus tip is inverted hat-shaped, 3 µm broad × 1.5 µm high, bluing intensely in Melzer’s reagent	(6–)8-spored, unitunicate, cylindrical, long-stipitate, 115–160 × 8–10 µm (x̄ = 2.5 × 3 μm) total length, with apical apparatus, 2–3 µm high, 2.8–3.2 µm wide (x̄ = 2.5 × 3 μm), appearing inverted trapezoid in optical longitudinal section, bluing in Melzer’s reagent.
Spores			
shape	oblong-fusoid, blunt or somewhat pointed at both ends	Several fusoid-inequilateral ascospores were observed, but these probably were not entirely mature.	globose to oval
color	brownish-black to dark brown	Light brown	hyaline with single, central guttule when young, becoming brown, ellipsoid with acute ends, smooth, thick-walled at maturity
inner contents	containing 1–3 droplets internally, germ slit not described	Not described, no germ slit was observed	containing a large central and two small polar guttules, with a conspicuous, S-shaped germ slit one-half to four-fifths spore length on the flattened side.
Size	12–17 × 4–6 µm	14.7–17 × 5.9–6.6 µm	12–17 × 5–7 µm
Conidia	in long chains, spherical or egg-shaped conidia 6–9 µm in size, catenulate, hyaline, spherical to ovoid, with a granular or warty ornamentation	Not described	Conidia 6–9 µm length, 3–5 µm width (x̄ = 8 × 4 µm, n = 25), produced singly on denticles, ellipsoid to ovoid, hyaline, smooth, unicellular.
Habit	Subterranean ()?	Not described	Epigeous
Specimen examined	New Caledonia, near Nouméa, subterranean()? on hills. June 1903. Le Rat.	New Caledonia: Le Rat no. 19, VI.1906, “Parasitic on old stumps of Casuarina” [translated from French] (FH);?Le Rat [as Penzigia compuncta (Jungh.) sterile]	China: Yunnan Province, Kunming, Luquan Yi and Miao Autonomous County, (HKAS148941); Xishuangbanna Dai Autonomous Prefecture (HKAS150824); Chuxiong Yi Autonomous Prefecture (HKAS150823); in subtropical and tropical forests.
Specimen deposited at	Lost	FH	HKAS

## 4 Discussion

Yunnan Province, situated in southwestern China, is recognized for its exceptional fungal diversity (Feng and Yang, 2018). Its varied topography, encompassing subtropical forests, alpine meadows, grasslands, and wetlands, creates a spectrum of ecological niches and microhabitats that support a rich mycota (Sun et al., 2017). Driven by complex landscapes, diverse floristic communities hosting numerous endemic species, and predominantly tropical to subtropical climates, Yunnan constitutes a biodiversity hotspot for fungi. While over 6,000 species have been documented, total fungal richness is estimated to potentially exceed 100,000 species, with frequent discoveries of novel taxa and genera continuing to highlight the region’s underexplored status (Senanayake et al., 2022). Research has extensively documented major phyla such as *Ascomycota* and *Basidiomycota*.

The Province hosts a significant diversity of ectomycorrhizal fungi, which form symbiotic associations with tree roots, particularly in temperate and subtropical forests (Liu et al., 2009; Dong et al., 2024; Yang et al., 2025; Wang et al., 2026). Commonly encountered genera include *Boletus*, *Ganoderma*, *Russula*, *Tricholoma*, *Tuber* (truffles), and *Xylaria.* These fungi play critical roles in nutrient cycling and forest ecosystem functioning. Yunnan is also notable for its edible fungi, especially *Morchella* species and truffles (genus *Tuber*), which hold substantial culinary and economic value both locally and globally. The utilization of wild edible mushrooms represents an important economic activity though it raises concurrent concerns regarding sustainable harvest and ecological impact. Thus, the diversity of edible fungi is a major focus of both ecological and economic research.

Up to now, very few species in *Xylariales* have been reported to produce massive stromata. The size of the stromata in *Xylariales* depends on the species, mode of life, and geographical location of collections. *Engleromyces goetzei* P.Henn. represents the largest species reported within the order *Xylariales* to date. *Engleromyces goetzei* produces subglobose, two-lobed stromata that are seated on and partially enveloping bamboo culms (Whalley et al., 2010). Gibson and Kimeria (1962) reported *Engleromyces goetzei* from North Kenya, where it forms subglobose stromata growing toward the tops of bamboo stems, about 30–40 ft. from the ground, varying considerably in size, from small growths weighing a few ounces to the largest with a diameter of nearly 30 cm and weighing around 3.2 kg. Meanwhile, *Engleromyces sinensis* M.A. Whalley, A. Khalil, T.Z. Wei, Y.J. Yao & Whalley, sampled in Yulong County in Yunnan Province, China, presented much smaller stromata that enveloped the bamboo culms and formed two lobes; the stromata were globose to subglobose, with sizes of 4.3–4.9 × 4–5.5 cm in width and 1.6–4 cm in height (Whalley et al., 2010). Most other species in Xylariales that morphologically resemble *Dianjunus rex* are *Daldinia concentrica* (Bolton) Cesati & de Notaris, *Xylaria hypoxylon* (L.) Grev., *X. cubensis* (Mont.) Fr., and *X. longipes* Nitschke. However, the majority of them produce black stromata. However, *Dianjunus rex* differs, apart from its larger size, in having less branched, pale to dark yellow stromata with blunt apex (Phillips et al., 2005).

Beyond its morphological distinctiveness, *Dianjunus rex* is phylogenetically isolated from all other known taxa in *Xylariales*. Divergence time estimates by Samarakoon et al. (2022) place the split between *Amphisphaeriales* and *Xylariales* at 154 (117–190) million years ago (MYA), with crown ages of 127 (92–165) MYA and 147 (111–184) MYA, respectively. Our analysis estimates the family *Dianjunaceae* diverged at approximately 65 (50–80) MYA (**Figure 2**), a timeframe consistent with the common family-level divergence trend of 50–150 MYA reported for fungi (Hyde et al., 2017). The phylogenomic topologies generated by the concatenation and summary-coalescent (ASTRAL) approaches were largely congruent, with one notable exception in a non-target lineage. Specifically, the phylogenetic position of *Stanjemonium grisellum* exhibited topological discordance between the concatenation tree and the two ASTRAL species trees (which were perfectly consistent with each other) (**Supplementary Data**). In the ASTRAL analyses, the local posterior probability (LPP) for this discordant node was 0.84 for the BUSCO dataset and dropped to 0.62 for the OrthoFinder dataset (**Figure 7**). This moderate-to-low support indicates historical gene tree conflicts, likely arising from widespread incomplete lineage sorting (ILS) or historical hybridization events within this non-target group. Crucially, despite this isolated background conflict, our core target topology was flawlessly and robustly preserved across all three distinct workflows. *Dianjunus* was consistently resolved as a sister group to the *Graphostromataceae* with maximum support (Bootstrap = 100 in the concatenation tree; LPP = 1.0 in both ASTRAL trees). This definitive consistency across diverse datasets and evolutionary models demonstrates that the taxonomic and evolutionary placement of our target family and genus is unequivocally stable (**Figure 7**). There are 432 monotypic fungal families, representing 35.4% of total known families in Kingdom Fungi (Hyde et al., 2024).

**Figure 7:**
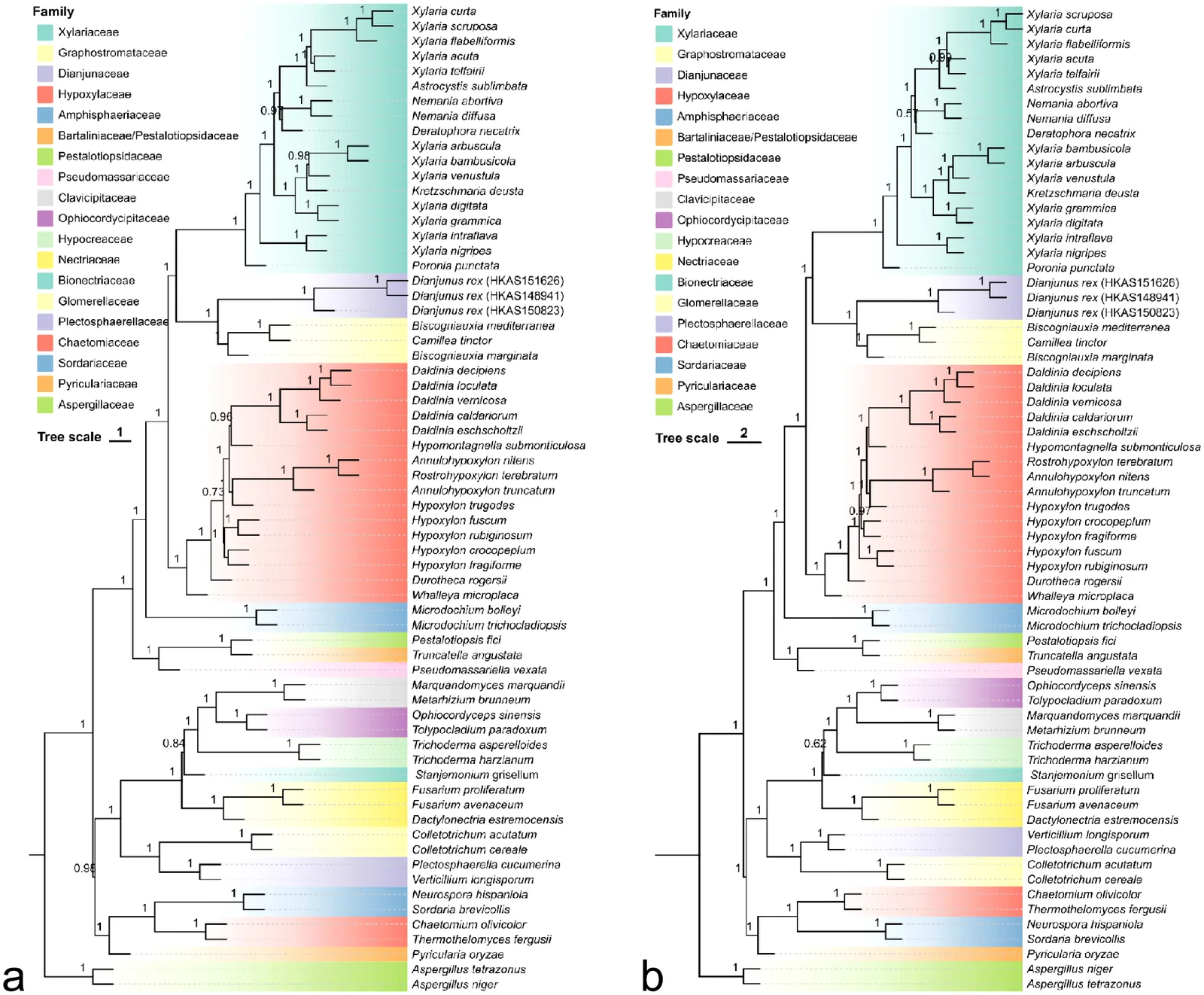
**(a)** Coalescent-Based Species Tree Inference Using BUSCO Dataset; **(b)** Alternative Phylogenetic Inference Based on OrthoFinder Dataset.

Species in *Graphostromataceae* show wide variations in anamorph morphology mainly described from sporulate cultures by nodulisporium-like conidial formation. However, sometimes other types of conidial formation have also been observed, such as sporothrix-like, rhinocladiella-like, virgariella-like, xylocladium-like, and periconiella-like (Ju and Rogers, 1996; Wendt et al., 2018). Our collections form a nodulisporium-like anamorph with periconiella-like branching patterns. The conidiophores are 15–25 µm long, with distantly packed branchlets bearing clusters of cylindrical, conidiogenous cells that are distinct from those of species in *Graphostromataceae*.

Comparative genomics revealed a conserved contraction of CAZyme genes (~220 genes) across all three *D. rex* strains compared to related saprobic *Xylariales* (>350 genes; Miyauchi et al., 2020). The most striking feature is the near-total depletion of pectin lyases (PL = 0-1, vs. 7–19 in related saprobes; **Figure 2**). In plant–fungus interactions, pectinases play a pivotal role in the initial breakdown of host plant cell walls, and their cleavage products can act as damage-associated molecular patterns (DAMPs) that trigger host immune responses (De Lorenzo et al., 2019). The loss of PL genes indicates a reduced genomic potential for pectin degradation, a feature consistent with genomic signatures of certain biotrophic or latent fungal endophytes (Mesny et al., 2021).

Concurrently, glycosyltransferases are proportionally enriched (GTs: ~26% of total CAZymes; **Figure 2**), which are primarily responsible for fungal cell wall biosynthesis. Because fruiting bodies of *D. rex* were observed exclusively post-host mortality, these combined traits support a working hypothesis of an endophyte-to-saprotroph lifestyle transition (Selosse et al., 2018). In this scenario, low metabolic maintenance costs of extracellular degradative enzymes, paired with sustained allocation to cell wall synthesis, may facilitate rapid carbon channeling into fungal biomass to form its massive stromata (**Figure 5a**). Such genomic adaptations accord with the dual ecological strategies widely observed in xylarialean endophytes, where genomic streamlining aligns with metabolic trade-offs between symbiotic latency and saprotrophic competence (Ren et al., 2022).

## 5 Conclusion

This study elucidates the taxonomic identity and phylogenetic relationships of an exceptional macromycete discovered in the subtropical and tropical forests of Yunnan Province, China. Through an integrative taxonomic approach combining detailed morphological examination, multi-locus phylogenetic analysis (ITS, LSU, *RPB2*, and *TUB2*), and phylogenomic data, the fungus is formally described as *Dianjunus rex* gen. et sp. nov. Phylogenetic reconstructions robustly place this novel taxon within the order *Xylariales*, where it forms a distinct, well-supported lineage sister to the family *Graphostromataceae*. This phylogenetic isolation, coupled with a unique suite of morphological characters, including massive, upright, fleshy stromata, a persistent powdery-fibrous surface, and a nodulisporium-like anamorph, is proposed to establish the new family *Dianjunaceae*. Divergence time estimation, calibrated using a genome-scale dataset, indicates that the *Dianjunaceae* lineage diverged during the early Paleocene, approximately 65 (50–80) million years ago. This timing coincides with the Cretaceous–Paleogene (K–Pg) boundary and the subsequent period of significant planetary ecological reorganization. The emergence of this morphologically distinct lineage in the aftermath of a major extinction event may reflect an adaptive radiation or the exploitation of newly available ecological niches during forest ecosystem recovery. The stromata of *D. rex*, reaching dimensions of up to 50 cm in height and 2.2 kg in weight, represent the largest stromata documented within the *Ascomycota*. This discovery significantly expands the known morphological disparity of the *Xylariales*, demonstrating that the evolution of gigantism in ascomycete fructifications has occurred independently in multiple lineages, including the stromata-forming *Xylariales* and the apothecia- or truffle-forming *Pezizales*. The discovery of *Dianjunus rex* and the *Dianjunaceae* underscores the profound and largely unexplored fungal diversity within East Asian forests, particularly in the biodiverse regions of southwestern China. It affirms the critical importance of continued mycological exploration integrated with modern molecular systematics, which remains essential for revealing hidden lineages, understanding the evolutionary trajectories of fungal morphology, and elucidating the complex history of Earth’s ecosystems.

## Data Availability

The datasets presented in this study can be found in online repositories. The names of the repository/repositories and accession number(s) can be found in the article/**Supplementary Material**.

## References

[B1] Bankevich A. Nurk S. Antipov D. Gurevich A. A. Dvorkin M. Kulikov A. S. . (2012). SPAdes: a new genome assembly algorithm and its applications to single-cell sequencing. J. Comput. Biol. 19, 455–477. doi: 10.1089/cmb.2012.0021 22506599 PMC3342519

[B2] Becerra-Hernández C. I. González D. De Luna E. Mena-Portales J. (2016). First report of pleoanamorphy in Gyrothrix verticiclada with an Idriella-like synanamorph. Cryptogam. Mycol. 37, 241–252. doi: 10.7872/crym/v37.iss2.2016.24142639091

[B3] Becker K. Stadler M. (2021). Recent progress in biodiversity research on the Xylariales and their secondary metabolism. J. Antibiot. 74, 1–23. doi: 10.1038/s41429-020-00376-0 33097836 PMC7732752

[B4] Becker K. Wongkanoun S. Wessel A.-C. Lambert C. Stadler M. (2020). Phylogenetic and chemotaxonomic studies confirm the affinities of Stromatoneurospora phoenix to the coprophilous Xylariaceae. J. Fungi 6, 144. doi: 10.3390/jof6030144 32842463 PMC7558325

[B5] Bhattacharya D. Lutzoni F. Reeb V. Simon D. M. Wilson R. Tehler A. (2000). Widespread occurrence of spliceosomal introns in the rDNA genes of ascomycetes. Persoonia 17, 1971–1984. doi: 10.1093/oxfordjournals.molbev.a026298 11110913

[B6] Birney E. Clamp M. Durbin R. (2004). GeneWise and genomewise. Genome Res. 14, 988–995. doi: 10.1101/gr.1865504 15123596 PMC479130

[B7] Cavalcante A. L. A. Negreiros A. M. P. Tavares M. B. de Oliveira E. J. da Silva G. F. (2020). Characterization of five new Monosporascus species: adaptation to environmental factors, pathogenicity to cucurbits and sensitivity to fungicides. J. Fungi 6, 169. doi: 10.3390/jof6030169 32927599 PMC7560037

[B8] Cedeño-Sanchez M. Schiefelbein R. Stadler M. Voglmayr H. (2023). Redisposition of apiosporous genera Induratia and Muscodor in the Xylariales, following the discovery of an authentic strain of Induratia apiospora. Bot. Stud. 64, 8. doi: 10.1186/s40529-023-00372-1 37052736 PMC10102272

[B9] Chen N. (2004). Using RepeatMasker to identify repetitive elements in genomic sequences. Curr. Protoc. Bioinf. 5, 4.10.1–4.10.14. doi: 10.1002/0471250953.bi0410s05 19274634

[B10] Chen S. Zhou Y. Chen Y. Gu J. (2018). fastp: an ultra-fast all-in-one FASTQ preprocessor. Bioinformatics 34, i884–i890. doi: 10.1093/bioinformatics/bty560 30423086 PMC6129281

[B11] Cheng X. Li W. Cai L. (2015). Molecular phylogeny of Ascotricha, including two new marine algae-associated species. Mycologia 107, 490–504. doi: 10.2307/26506910 25724998

[B12] Chen Y. P. Su P. W. Yang C. H. Chen C. W. (2023). Phylogenomics and diversification of sordariomycetes. Mycosphere 14, 414–451. doi: 10.5943/mycosphere/14/1/5

[B13] Crous P. W. Schumacher R. K. Akulov A. Thangavel R. Hyde K. D. (2019). New and interesting fungi 2. Persoonia 43, 57–134. doi: 10.3114/fuse.2019.03.06 32467898 PMC7235984

[B14] Crous P. W. Schumacher R. K. Wingfield M. J. Lombard L. Giraldo A. Christensen M. . (2015). Fungal systematics and evolution: FUSE 1. Sydowia 67, 81–118. doi: 10.12905/0380.sydowia67-2015-0081

[B15] Crous P. W. Shivas R. G. Quaedvlieg W. van der Bank M. Zhang Y. Summerell B. A. . (2014). Fungal Planet description sheets: 214–280. Persoonia 32, 184–306. doi: 10.3767/003158514X682395 25264390 PMC4150077

[B16] Crous P. W. Wingfield M. J. Chooi Y. H. Gilbert D. Lacey E. Pitt J. I. (2020). Fungal Planet description sheets: 1042–1111. Persoonia 44, 301–459. doi: 10.3767/persoonia.2020.44.11 33116344 PMC7567971

[B17] Dai D. Q. Bahkali A. H. Li Q. R. Bhat D. J. Zhao R. L. Hyde K. D. . (2014). Vamsapriya (Xylariaceae) re-described, with two new species and molecular sequence data. Cryptogam. Mycol. 35, 339–357. doi: 10.7872/crym.v35.iss4.2014.3390020

[B18] Dai D. Q. Phookamsak R. Wijayawardene N. N. Li W. J. Bhat D. J. Xu J. . (2016). Bambusicolous fungi. Fungal Divers. 82, 1–105. doi: 10.1007/s13225-016-0367-830311153

[B19] Daranagama D. A. Camporesi E. Tian Q. Hyde K. D. (2015). Anthostomella is polyphyletic comprising several genera in Xylariaceae. Fungal Divers. 73, 203–238. doi: 10.1007/s13225-015-0329-630311153

[B20] Dayarathne M. C. Wanasinghe D. N. Devadatha B. Jones E. B. G. Hyde K. D. (2020). Modern taxonomic approaches to identifying diatrypaceous fungi from marine habitats, with a novel genus Halocryptovalsa Dayarathne & K.D. Hyde, gen. nov. Cryptogam. Mycol. 41, 21–67. doi: 10.5252/cryptogamie-mycologie2020v41a3

[B21] de Almeida D. A. C. Gusmão L. F. P. Miller A. N. (2016). Taxonomy and molecular phylogeny of Diatrypaceae (Ascomycota, Xylariales) species from the Brazilian semi-arid region, including four new species. Mycol. Prog. 15, 53. doi: 10.1007/s11557-016-1194-830311153

[B22] Delgado G. Miller A. N. Crous P. W. Hyde K. D. (2025). New genera, species, combinations, and synonyms of saprobic Dothideomycetes and Sordariomycetes from subtropical Texas, USA. Mycologia 117, 1104–1147. doi: 10.1080/00275514.2025.2540233 40906549

[B23] De Lorenzo G. Ferrari S. Moira G. Mattei B. Cervone F. (2019). Cell wall traits that influence plant development, immunity, and bioconversion. Plant J. 97, 134–147. doi: 10.1111/tpj.14196 30548980

[B24] Dissanayake L. S. Maharachchikumbura S. S. N. Mortimer P. E. (2021). Acrocordiella yunnanensis sp. nov. (Requienellaceae, Xylariales) from Yunnan, China. Phytotaxa 487, 103–113. doi: 10.11646/phytotaxa.487.2.1

[B25] Dissanayake A. J. Shang Q. J. Chi M. F. Hyde K. D. Zhao R. L. (2025). Insights into the taxonomy and phylogeny of Diatrypaceae. Fungal Divers. 134, 461–634. doi: 10.1007/s13225-025-00563-030311153

[B26] Doilom M. Hyde K. D. Phookamsak R. Bhat D. J. Jones E. B. G. Maharachchikumbura S. S. N. . (2018). Mycosphere Notes 225–274: types and other specimens of some genera of Ascomycota. Mycosphere 9, 647–754. doi: 10.5943/mycosphere/9/4/3

[B27] Dong J. H. Li Q. Yuan Q. Luo Y. X. Zhang X. C. Dai Y. F. . (2024). Species diversity, taxonomy, molecular systematics and divergence time of wood-inhabiting fungi in Yunnan-Guizhou Plateau, Asia. Mycosphere 15, 1110–1293. doi: 10.5943/mycosphere/15/1/10

[B28] Duong L. Lumyong S. Hyde K. D. Whalley M. A. (2004). Emarcea castanopsidicola gen. et sp. nov. from Thailand, a new xylariaceous taxon based on morphology and DNA sequences. Stud. Mycol. 50, 253–260.

[B29] Feng B. Yang Z. (2018). Studies on diversity of higher fungi in Yunnan, southwestern China: A review. Plant Divers. 40, 165–171. doi: 10.1016/j.pld.2018.07.001 30740561 PMC6137262

[B30] Flynn J. M. Hubley R. Goubert C. Rosen J. Clark A. G. Feschotte C. . (2020). RepeatModeler2 for automated genomic discovery of transposable element families. Proc. Natl. Acad. Sci. U.S.A. 117, 201921046. doi: 10.1073/pnas.1921046117 32300014 PMC7196820

[B31] Fournier J. Lechat C. Courtecuisse R. (2018). The genera kretzschmariella and nemania (Xylariaceae) in Guadeloupe and Martinique (French West Indies). Ascomycete.org 10, 1–47. doi: 10.25664/art-0226

[B32] Fournier J. Lechat C. Courtecuisse R. (2020). The genus Xylaria sensu lato (Xylariaceae) in Guadeloupe and Martinique (French West Indies) III. Taxa with slender upright Stromata. Ascomycete.org 12 (3), 81–164. doi: 10.25664/ART-0302

[B33] Fournier J. Stadler M. Hyde K. D. Wongkanoun S. (2010). The new genus Rostrohypoxylon and two new Annulohypoxylon species from Northern Thailand. Fungal Divers. 40, 23–36. doi: 10.1007/s13225-010-0026-430311153

[B34] Franco M. E. E. Nickerson M. N. Bowen B. P. Louie K. B. Wang Y. Northen T. R. . (2025). Hyperdiverse, bioactive, and interaction-specific metabolites produced only in co-culture suggest diverse competitors may fuel secondary metabolism of xylarialean fungi. mSystems 10, e00468-25. doi: 10.1128/msystems.00468-25 40488491 PMC12282070

[B35] Franco M. E. E. Wisecaver J. H. Arnold A. E. Ju Y. M. Slot J. C. Ahrendt S. . (2022). Ecological generalism drives hyperdiversity of secondary metabolite gene clusters in xylarialean endophytes. New Phytol. 233, 1317–1330. doi: 10.1111/nph.17873 34797921

[B36] Gao J. Liu S. Zou S. Zhang Y. (2023). An epitype of Engleromyces sinensis designated here. J. Edible Fungi 30, 102–110. doi: 10.16488/j.cnki.1005-9873.2023.02.011

[B37] Gibson I. A. Kimeria J. (1962). A short note on Engleromyces goetzei P. Henn. East Afr. Agric. For. J. 28, 61–63. doi: 10.1080/00128325.1962.1166184337339054

[B38] Hall T. A. (1999). BioEdit: A user-friendly biological sequence alignment editor and analysis program for Windows 95/98/NT. Nucleic Acids Symp. Ser. 41, 95–98. doi: 10.14601/Phytopathol_Mediterr-14998u1.29

[B39] Hartley J. M. H. (2020). Surveillance and isolation of Rosellinia necatrix in South African avocado orchards Msc Dissertation, University of Pretoria, Pretoria. Available online at: http://hdl.handle.net/2263/75181 (Accessed April 25, 2026).

[B40] Hennings P. C. (1903). Über die in Gebäuden auftretenden wichtigsten holzbewohnenden Schwämme. Hedwigia 42, 178–191.

[B41] Hernández-Restrepo M. Gené J. Castañeda-Ruiz R. F. Mena-Portales J. Guarro J. Hyde K. D. (2017). Phylogeny of saprobic microfungi from Southern Europe. Stud. Mycol. 86, 53–97. doi: 10.1016/j.simyco.2017.05.002 28626275 PMC5470572

[B42] Hernández-Restrepo M. Groenewald J. Z. Crous P. W. (2016). Taxonomic and phylogenetic re-evaluation of Microdochium, Monographella and Idriella. Persoonia 36, 57–82. doi: 10.3767/003158516x688676 27616788 PMC4988376

[B43] Hsieh H. Lin C. R. Fang M. J. Rogers J. D. Fournier J. Lechat C. . (2010). Phylogenetic status of Xylaria subgenus Pseudoxylaria among taxa of the subfamily Xylarioideae (Xylariaceae) and phylogeny of the taxa involved in the subfamily. Persoonia 54, 957–969. doi: 10.1016/j.ympev.2009.12.015 20035889

[B44] Hyde K. D. Dong Y. Phookamsak R. Jeewon R. Bhat D. J. Jones E. B. G. . (2020a). Fungal diversity notes 1151–1276: taxonomic and phylogenetic contributions on genera and species of fungal taxa. Fungal Divers. 100, 5–277. doi: 10.1007/s13225-020-00439-530311153

[B45] Hyde K. D. Noorabadi M. T. Thiyagaraja V. He M. Q. Johnston P. R. Jones E. B. G. (2024). The 2024 Outline of Fungi and fungus-like taxa. Mycosphere 15, 5146–6239. doi: 10.5943/mycosphere/15/1/25

[B46] Hyde K. D. Norphanphoun C. Abreu V. P. Bazzicalupo A. Beimforde C. Bittencourt F. . (2017). Fungal diversity notes 603–708: taxonomic and phylogenetic notes on genera and species. Fungal Divers. 87, 1–235. doi: 10.1007/s13225-017-0391-330311153

[B47] Hyde K. D. Norphanphoun C. Maharachchikumbura S. S. N. Bhat D. J. Jones E. B. G. Bundhun D. . (2020b). Refined families of sordariomycetes. Mycosphere 11, 305–1059. doi: 10.5943/mycosphere/11/1/7

[B48] Jaklitsch W. M. Fournier J. Rogers J. D. Voglmayr H. (2014). Phylogenetic and taxonomic revision of Lopadostoma. Persoonia 32, 52–82. doi: 10.3767/003158514x679272 25264383 PMC4150080

[B49] Jaklitsch W. M. Gardiennet A. Voglmayr H. (2016). Resolution of morphology-based taxonomic delusions: Acrocordiella, Basiseptospora, Blogiascospora, Clypeosphaeria, Hymenopleella, Lepteutypa, Pseudapiospora, Requienella, Seiridium and Strickeria. Persoonia 37, 82–105. doi: 10.3767/003158516x690475 28100927 PMC5238940

[B50] Jaklitsch W. M. Voglmayr H. (2012). Phylogenetic relationships of five genera of Xylariales and Rosasphaeria gen. nov. (Hypocreales). Fungal Divers. 52, 75–98. doi: 10.1007/s13225-011-0104-230311153

[B51] Jayanetti D. R. Yue Q. Bills G. F. Strobel G. A. (2014). Hypocoprins a–c: New sesquiterpenoids from the coprophilous fungus Hypocopra rostrata. J. Nat. Prod. 78, 396–401. doi: 10.1021/np5007718 25549014 PMC4380195

[B52] Johnston P. R. Rogers J. D. Park D. Jones E. B. G. (2016). Entalbostroma erumpens gen. et sp. nov. (Xylariaceae) from Phormium in New Zealand. Mycotaxon 131, 765–771. doi: 10.5248/131.76530528588

[B53] Ju Y. Rogers J. D. (1996). “ A revision of the genus Hypoxylon,” in Mycologia Memoir ( APS Press, St. Paul), 1–365.

[B54] Karunarathna A. Działak P. Jayawardena R. S. Hyde K. D. (2021). A novel addition to the Pezizellaceae (Rhytismatales, Ascomycota). Phytotaxa 480, 251–261. doi: 10.11646/phytotaxa.480.3.4

[B55] Katoh K. Misawa K. Kuma K. Miyata T. (2002). MAFFT: a novel method for rapid multiple sequence alignment based on fast Fourier transform. Nucleic Acids Res. 30, 3059–3066. doi: 10.1093/nar/gkf436 12136088 PMC135756

[B56] Katoh K. Rozewicki J. Yamada K. D. (2019). MAFFT online service: multiple sequence alignment, interactive sequence choice and visualization. Brief. Bioinform. 20, 1160–1166. doi: 10.1093/bib/bbx108 28968734 PMC6781576

[B57] Kim D. Langmead B. Salzberg S. L. (2015). HISAT: a fast spliced aligner with low memory requirements. Nat. Methods 12, 357–360. doi: 10.1038/nmeth.3317 25751142 PMC4655817

[B58] Klaysuban A. Sakayaroj J. Jones E. B. G. (2014). An additional marine fungal lineage in the Diatrypaceae, Xylariales: Pedumispora rhizophorae. Bot. Mar. 57, 413–420. doi: 10.1515/bot-2014-001731755547

[B59] Konta S. Maharachchikumbura S. S. N. Senanayake I. C. Hyde K. D. (2020). A new genus Allodiatrype, five new species and a new host record of diatrypaceous fungi from palms (Arecaceae). Mycosphere 11, 239–268. doi: 10.5943/mycosphere/11/1/4

[B60] Kornsakulkarn J. Saepua S. Suvannakad R. Isaka M. (2017). Cytotoxic tropolones from the fungus Nemania sp. BCC 30850. Tetrahedron 73, 3505–3512. doi: 10.1016/j.tet.2017.05.03042574925

[B61] Kumar S. Stecher G. Suleski M. Hedges S. B. (2017). TimeTree: a resource for timelines, timetrees, and divergence times. Mol. Biol. Evol. 34, 1812–1819. doi: 10.1093/molbev/msx116 28387841

[B62] Lambert C. Wendt L. Hladki A. I. Sir E. B. Stadler M. (2019). Hypomontagnella (Hypoxylaceae): a new genus segregated from Hypoxylon by a polyphasic taxonomic approach. Mycol. Prog. 18, 187–201. doi: 10.1007/s11557-018-1452-z30311153

[B63] Li W. J. McKenzie E. H. C. Liu J. K. Bhat D. J. Dai D. Q. Hyde K. D. (2020). Taxonomy and phylogeny of hyaline-spored coelomycetes. Fungal Divers. 100, 279–801. doi: 10.1007/s13225-020-00440-y30311153

[B64] Li D. W. Schultes N. P. Chen J. Y. Hyde K. D. (2017). Circinotrichum sinense, a new asexual fungus from Hubei, China. Botany 95, 1099–1108. doi: 10.1139/cjb-2017-013234819996

[B65] Liu J. K. Hyde K. D. Jones E. Y. Ariyawansa H. A. Bhat D. J. Boonmee S. . (2015). Fungal diversity notes 1–110: taxonomic and phylogenetic contributions to fungal species. Fungal Divers. 72, 1–197. doi: 10.1007/s13225-015-0324-y30311153

[B66] Liu P. G. Wang X. H. Yu F. Q. Yang Z. L. (2009). Fungous kingdom: yunnan of China and her ectomycorrhizal macrofungal species diversity. Acta Bot. Yunnanica 16, 15–20.

[B67] Liu Y. J. Whelen S. Hall B. D. (1999). Phylogenetic relationships among ascomycetes: evidence from an RNA polymerase II subunit. Mol. Biol. Evol. 16, 1799–1808. doi: 10.1093/oxfordjournals.molbev.a026092 10605121

[B68] Lloyd C. G. (1917). “ Synopsis of some genera of the large Pyrenomycetes: Camillea, Thamnomyces, Engleromyces,” in Hard Press Publishing. (Ohio: Cincinnati) 1–39.

[B69] Long Q. Liu L. L. Zhang X. Hyde K. D. (2019). Contributions to species of Xylariales in China-1. Durotheca species. Mycol. Prog. 18, 495–510. doi: 10.1007/s11557-018-1458-630311153

[B70] Ma X. Chomnunti P. Doilom M. Hyde K. D. (2022). Multigene phylogeny reveals endophytic Xylariales novelties from Dendrobium species from southwestern China and northern Thailand. J. Fungi 8, 248. doi: 10.3390/jof8030248 35330250 PMC8955275

[B71] Maharachchikumbura S. S. N. Hyde K. D. Perera R. H. Jones E. B. G. (2018). Acrocordiella omanensis sp. nov. (Requienellaceae, Xylariales) from the Sultanate of Oman. Phytotaxa 338, 294–294. doi: 10.11646/phytotaxa.338.3.7

[B72] Mapook A. Hyde K. D. McKenzie E. H. C. Jones E. B. G. Bhat D. J. (2020). Taxonomic and phylogenetic contributions to fungi associated with the invasive weed Chromolaena odorata (Siam weed). Fungal Divers. 101, 1–175. doi: 10.1007/s13225-020-00444-830311153

[B73] Marincowitz S. Crous P. W. Groenewald J. Z. Wingfield M. J. (2008). “ Microfungi occurring on Proteaceae in the fynbos,” in Cbs-Knaw Fungal Biodiversity Centre. (Utrecht: CBS-KNAW Fungal Biodiversity Centre).

[B74] Mesny F. Miyauchi S. Thiergart T. Pickel B. Atanasova L. Karlsson M. . (2021). Genetic determinants of endophytism in the *Arabidopsis* root mycobiome. Nat. Commun. 12, 7227. doi: 10.1038/s41467-021-27479-y 34893598 PMC8664821

[B75] Mirabolfathy M. Ju Y. M. Hsieh H. M. Asgari B. (2013). Obolarina persica sp. nov. associated with dying Quercus in Iran. Mycoscience 54, 315–320. doi: 10.1016/j.myc.2012.11.00342574925

[B76] Miyauchi S. Kiss E. Kuo A. Drula E. Kohler A. Sánchez-García M. . (2020). Large-scale genome sequencing of mycorrhizal fungi provides insights into the early evolution of symbiotic traits. Nat. Commun. 11, 5125. doi: 10.1038/s41467-020-18795-w 33046698 PMC7550596

[B77] Nylander J. A. A. (2004). “ MrModeltest version 2. Program distributed by the author,” in Evolutionary Biology Centre, Uppsala University. (Uppsala: Evolutionary Biology Centre).

[B78] O'Donnell K. Cigelnik E. (1997). Two divergent intragenomic rDNA ITS2 types within a monophyletic lineage of the fungus Fusarium are nonorthologous. Mol. Phylogenet. Evol. 7, 103–116. doi: 10.1006/mpev.1996.0376 9007025

[B79] Okane I. Nakagiri A. Ito T. (2001). Surculiseries rugispora gen. et sp. nov. a new endophytic mitosporic fungus from leaves of Bruguiera gymnorrhiza. Mycoscience 42, 115–122. doi: 10.1007/bf0246398430311153

[B80] Pažoutová S. Šrůtka P. Holuša J. Fournier J. (2012). Liberomyces gen. nov. with two new species of endophytic coelomycetes from broadleaf trees. Mycologia 104, 198–210. doi: 10.3852/11-081 21937729

[B81] Peláez F. González V. Platas G. Martín J. (2008). Molecular phylogenetic studies within the family Xylariaceae based on ribosomal DNA sequences. Fungal Divers. 31, 111–134.

[B82] Perera R. H. Maharachchikumbura S. S. N. Jones E. B. G. Hyde K. D. (2017). Delonicicola siamense gen. & sp. nov. (Delonicicolaceae fam. nov. Delonicicolales ord. nov.), a saprobic species from Delonix regia seed pods. Cryptogam. Mycol. 38, 321–340. doi: 10.7872/crym/v38.iss3.2017.32142639091

[B83] Peršoh D. Melcher M. Graf K. Fournier J. (2009). Molecular and morphological evidence for the delimitation of Xylaria hypoxylon. Mycologia 101, 256–268. doi: 10.3852/08-108 19397200

[B84] Phillips R. Kibby G. Foy N. Jordan M. (2005). “ Mushrooms and other fungi of North America, Vol. 2,” in Firefly Books. (Ontario: Firefly Books Ltd), 1–319.

[B85] Phookamsak R. Hyde K. D. Jeewon R. Bhat D. J. Jones E. B. G. Maharachchikumbura S. S. N. . (2019). Fungal diversity notes 929–1035: taxonomic and phylogenetic contributions on genera and species of fungi. Fungal Divers. 95, 1–273. doi: 10.1007/s13225-019-00421-w30311153

[B86] Phukhamsakda C. McKenzie E. H. C. Phillips A. J. L. Jones E. B. G. (2020). Microfungi associated with Clematis (Ranunculaceae) with an integrated approach to delimiting species boundaries. Fungal Divers. 102, 1–203. doi: 10.1007/s13225-020-00448-430311153

[B87] Porebski S. Bailey L. G. Baum B. R. (1997). Modification of a CTAB DNA extraction protocol for plants containing high polysaccharide and polyphenol components. Plant Mol. Biol. Rep. 15, 8–15. doi: 10.1007/bf0277210830311153

[B88] Rambaut A. (2012). FigTree, version 1.4. Available online at: http://tree.bio.ed.ac.uk/software/figtree (Accessed February 20, 2026).

[B89] Rambaut A. Drummond A. J. (2007). Tracer v1.4: MCMC Trace Analyses Tool. Available online at: http://beast.community/tracer (Accessed February 20, 2026).

[B90] Ren W. Yuan Y. Peng J. Mutti L. Jiang X. (2022). The function and clinical implication of circular RNAs in lung cancer. Front. Oncol. 12, 862602. doi: 10.3389/fonc.2022.862602 36338714 PMC9629004

[B91] Rogers J. D. (1979). The Xylariaceae: systematic, biological and evolutionary aspects. Mycologia 71, 1–42. doi: 10.1080/00275514.1979.1202098437339054

[B92] Rogers J. D. (1981). Sarcoxylon and entonaema (Xylariaceae). Mycologia 73, 28–61. doi: 10.1080/00275514.1981.1202131937339054

[B93] Rolshausen P. E. Mahoney N. E. Molyneux R. J. Gubler W. D. (2006). A reassessment of the species concept in Eutypa lata, the causal agent of Eutypa-dieback of grapevine. Phytopathology 96, 369–377. doi: 10.1094/phyto-96-0369 18943418

[B94] Ronquist F. Teslenko M. van der Mark P. Ayres D. L. Darling A. Höhna S. . (2012). MrBayes 3.2: efficient bayesian phylogenetic inference and model choice across a large model space. Syst. Biol. 61, 539–542. doi: 10.1093/sysbio/sys029 22357727 PMC3329765

[B95] Samarakoon M. C. Hyde K. D. Maharachchikumbura S. S. N. Jones E. B. G. (2022). Taxonomy, phylogeny, molecular dating and ancestral state reconstruction of Xylariomycetidae (Sordariomycetes). Fungal Divers. 112, 1–88. doi: 10.1007/s13225-021-00495-530311153

[B96] Samarakoon M. C. Hyde K. D. Promputtha I. Ariyawansa H. A. Jones E. B. G. (2016). Evolution of xylariomycetidae (Ascomycota: Sordariomycetes). Mycosphere 7, 1746–1761. doi: 10.5943/mycosphere/7/11/9

[B97] Samarakoon M. C. Thongbai B. Hyde K. D. Stadler M. (2020). Elucidation of the life cycle of the endophytic genus Muscodor and its transfer to Induratia in Induratiaceae fam. nov. based on a polyphasic taxonomic approach. Fungal Divers. 101, 177–210. doi: 10.1007/s13225-020-00443-930311153

[B98] Schoch C. L. Seifert K. A. Huhndorf S. Robert V. Spouge J. L. Levesque C. A. . (2012). Nuclear ribosomal internal transcribed spacer (ITS) region as a universal DNA barcode marker for Fungi. Proc. Natl. Acad. Sci. U.S.A. 109, 6241–6246. doi: 10.1073/pnas.1117018109 22454494 PMC3341068

[B99] Selosse M. A. Schneider-Maunoury L. Martos F. (2018). Time to re-think fungal ecology? Fungal ecological niches are often prejudged. New Phytol. 217, 968–972. doi: 10.1111/nph.14983 29334598

[B100] Senanayake I. C. Jeewon R. Hyde K. D. Jones E. B. G. (2020a). Taxonomy and phylogeny of Leptosillia cordylinea sp. nov. from China. Phytotaxa 435, 203–212. doi: 10.11646/phytotaxa.435.3.1

[B101] Senanayake I. C. Maharachchikumbura S. S. N. Hyde K. D. Jones E. B. G. (2015). Towards unraveling relationships in xylariomycetidae (Sordariomycetes). Fungal Divers. 73, 73–144. doi: 10.1007/s13225-015-0340-y30311153

[B102] Senanayake I. C. Pem D. Rathnayaka A. R. Jones E. B. G. (2022). Predicting global numbers of teleomorphic ascomycetes. Fungal Divers. 114, 237–278. doi: 10.1007/s13225-022-00498-w30311153

[B103] Senanayake I. C. Rathnayaka A. R. Marasinghe D. S. Jones E. B. G. (2020b). Morphological approaches in studying fungi: collection, examination, isolation, sporulation and preservation. Mycosphere 11, 2678–2754. doi: 10.5943/mycosphere/11/1/20

[B104] Senwanna C. Phookamsak R. Doilom M. Hyde K. D. (2017). Novel taxa of diatrypaceae from para rubber (Hevea brasiliensis) in northern Thailand; introducing a novel genus Allocryptovalsa. Mycosphere 8, 1835–1855. doi: 10.5943/mycosphere/8/10/9

[B105] Shang Q. J. Hyde K. D. Jeewon R. Jones E. B. G. (2018). Morpho-molecular characterization of Peroneutypa (Diatrypaceae, Xylariales) with two novel species from Thailand. Phytotaxa 356, 1–18. doi: 10.11646/phytotaxa.356.1.1

[B106] Shang Q.-J. Hyde K. D. Phookamsak R. Jones E. B. G. (2017). Diatrypella tectonae and Peroneutypa mackenziei sp. nov. (Diatrypaceae) from northern Thailand. Mycol. Prog. 16, 463–476. doi: 10.1007/s11557-017-1294-030311153

[B107] Silvestro D. Michalak I. (2012). raxmlGUI: a graphical front-end for RAxML. Org. Divers. Evol. 12, 335–337. doi: 10.1007/s13127-011-0056-030311153

[B108] Simão F. A. Waterhouse R. M. Ioannidis P. Kriventseva E. V. Zdobnov E. M. (2015). BUSCO: assessing genome assembly and annotation completeness with single-copy orthologs. Bioinformatics 31, 3210–3212. doi: 10.1093/bioinformatics/btv351 26059717

[B109] Sir E. B. Becker K. Lambert C. Stadler M. (2019). Observations on Texas Hypoxylons, including two new Hypoxylon species and widespread environmental isolates of the H. croceum complex identified by a polyphasic approach. Mycologia 111, 832–856. doi: 10.1080/00275514.2019.1637705 31460851

[B110] Spatafora J. W. Sung G. H. Johnson D. Hesse C. O'Rourke B. Serdani M. . (2006). A five-gene phylogeny of Pezizomycotina. Mycologia 98, 1018–1028. doi: 10.3852/mycologia.98.6.1018 17486977

[B111] Stadler M. (2011). Importance of secondary metabolites in the Xylariaceae as parameters for. Curr. Res. Environ. Appl. Mycol. 1, 75–133. doi: 10.5943/cream/1/2/142235046

[B112] Stadler M. Fournier J. Gardt S. Peršoh D. (2010). The phylogenetic position of Rhopalostroma as inferred from a polythetic approach. Persoonia 25, 11–21. doi: 10.3767/003158510x524231 21339963 PMC3028513

[B113] Stadler M. Læssøe T. Fournier J. Decock C. (2014). A polyphasic taxonomy of Daldinia (Xylariaceae). Stud. Mycol. 77, 1–143. doi: 10.3114/sim0016 24790283 PMC3953824

[B114] Stamatakis A. Alachiotis N. (2010). Time and memory efficient likelihood-based tree searches on phylogenomic alignments with missing data. Bioinformatics 26, i132–i139. doi: 10.1093/bioinformatics/btq205 20529898 PMC2881390

[B115] Stanke M. Keller O. Gunduz I. Hayes A. Waack S. Morgenstern B. (2006). AUGUSTUS: ab initio prediction of alternative transcripts. Nucleic Acids Res. 34, W435–W439. doi: 10.1093/nar/gkl200 16845043 PMC1538822

[B116] Sun H. Zhang J. W. Deng T. Boufford D. E. (2017). Origins and evolution of plants diversity in the Hengduan Mountains, China. Plant Divers. 39, 161–166. doi: 10.1016/j.pld.2018.07.001 30159507 PMC6112316

[B117] Tennakoon D. S. Kuo C. H. Maharachchikumbura S. S. N. Hyde K. D. (2021). Taxonomic and phylogenetic contributions to Celtis formosana, Ficus ampelas, F. septica, Macaranga tanarius and Morus australis leaf litter inhabiting microfungi. Fungal Divers. 108, 1–215. doi: 10.1007/s13225-021-00474-w30311153

[B118] Thitla T. Monkai J. Meng W. Hyde K. D. (2025). Two new species of Penicillium and a new genus in Xylariomycetidae from the forest dump-sites in Chiang Mai, Thailand. MycoKeys 116, 275–301. doi: 10.3897/mycokeys.116.150635 40337075 PMC12056519

[B119] Thiyagaraja V. Senanayake I. C. Wanasinghe D. N. Hyde K. D. (2019). Phylogenetic and morphological appraisal of Diatrype lijiangensis sp. nov. (Diatrypaceae, Xylariales) from China. Asian J. Mycol. 2, 198–208. doi: 10.5943/ajom/2/1/10

[B120] Tibpromma S. Daranagama D. A. Boonmee S. Hyde K. D. (2017). Anthostomelloides krabiensis gen. et sp. nov. (Xylariaceae) from Pandanus odorifer (Pandanaceae). Turk. J. Bot. 41, 107–116. doi: 10.3906/bot-1606-45

[B121] Triebel D. Peršoh D. Wollweber H. Fournier J. (2005). Phylogenetic relationships among Daldinia, Entonaema, and Hypoxylon as inferred from ITS nrDNA analyses of Xylariales. Nova Hedwigia 80, 25–43. doi: 10.1127/0029-5035/2005/0080-0025

[B122] Trouillas F. P. Gubler W. D. (2010). Host range, biological variation, and phylogenetic diversity of Eutypa lata in California. Phytopathology 100, 1048–1056. doi: 10.1094/phyto-02-10-0040 20839940

[B123] Trouillas F. P. Hand F. P. Inderbitzin P. Gubler W. D. (2015). The genus Cryptosphaeria in the western United States: taxonomy, multilocus phylogeny and a new species, C. multicontinentalis. Mycologia 107, 1304–1313. doi: 10.3852/15-115 26354808

[B124] Trouillas F. P. Pitt W. M. Sosnowski M. R. Gubler W. D. (2011). Taxonomy and DNA phylogeny of Diatrypaceae associated with Vitis vinifera and other woody plants in Australia. Fungal Divers. 49, 203–223. doi: 10.1007/s13225-011-0094-030311153

[B125] Trouillas F. P. Úrbez-Torres J. R. Gubler W. D. (2010). Diversity of diatrypaceous fungi associated with grapevine canker diseases in California. Mycologia 102, 319–336. doi: 10.3852/08-185 20361500

[B126] Úrbez-Torres J. R. Adams P. Kamas J. Gubler W. D. Leavitt G. (2009). Identification, incidence, and pathogenicity of fungal species associated with grapevine dieback in Texas. Am. J. Enol. Vitic. 60, 497–507. doi: 10.5344/ajev.2009.60.4.497

[B127] Vilgalys R. Hester M. (1990). Rapid genetic identification and mapping of enzymatically amplified ribosomal DNA from several Cryptococcus species. J. Bacteriol. 172, 4238–4246. doi: 10.1128/jb.172.8.4238-4246.1990 2376561 PMC213247

[B128] Voglmayr H. Beenken L. (2020). Linosporopsis, a new leaf-inhabiting scolecosporous genus in Xylariaceae. Mycol. Prog. 19, 205–222. doi: 10.1007/s11557-020-01559-7 32104168 PMC7008769

[B129] Voglmayr H. Friebes G. Gardiennet A. Jaklitsch W. M. (2018). Barrmaelia and Entosordaria in Barrmaeliaceae (fam. nov. Xylariales) and critical notes on Anthostomella-like genera based on multigene phylogenies. Mycol. Prog. 17, 155–177. doi: 10.1007/s11557-017-1329-6 29456465 PMC5801398

[B130] Vu D. Groenewald M. de Vries M. Gehrmann T. Stielow B. Eberhardt U. . (2019). Large-scale generation and analysis of filamentous fungal DNA barcodes boosts coverage for kingdom fungi and reveals thresholds for fungal species and higher taxon delimitation. Stud. Mycol. 92, 135–154. doi: 10.1016/j.simyco.2018.05.001 29955203 PMC6020082

[B131] Wanasinghe D. N. Phukhamsakda C. Hyde K. D. Jones E. B. G. (2018). Fungal diversity notes 709–839: taxonomic and phylogenetic contributions to fungal taxa with an emphasis on fungi on Rosaceae. Fungal Divers. 89, 1–236. doi: 10.1007/s13225-018-0395-730311153

[B132] Wang L. He S. Y. Lambert C. Zhu Y. G. Zhang J. L. Yang Y. . (2026). Comprehensive morphological and phylogenetic analyses of *Hymenochaetales* (Basidiomycota) unveil one new genus and twenty-sixnovel wood-inhabiting species from southwestern China. Persoonia 56, 328–380. doi: 10.3114/persoonia.2026.56.05 42524241 PMC13409181

[B133] Wendt L. Sir E. B. Kuhnert E. Stadler M. (2018). Resurrection and emendation of the Hypoxylaceae, recognised from a multigene phylogeny of the Xylariales. Mycol. Prog. 17, 115–154. doi: 10.1007/s11557-017-1311-330311153

[B134] Whalley M. A. Khalil A. M. A. Wei T. Z. Wang Y. (2010). A new species of Engleromyces from China, a second species in the genus. Mycotaxon 112, 317–323. doi: 10.5248/112.317

[B135] White T. J. Bruns T. Lee S. Taylor J. (1990). “ Amplification and direct sequencing of fungal ribosomal RNA genes for phylogenetics,” in PCR Protocols: A Guide to Methods and Applications. Eds. Innis M. A. Gelfand D. H. Sninsky J. J. White T. J. ( Academic Press Inc, New York), 315–322.

[B136] Wittstein K. Cordsmeier A. Lambert C. Stadler M. (2020). Identification of Rosellinia species as producers of cyclodepsipeptide PF1022 A and resurrection of the genus Dematophora as inferred from polythetic taxonomy. Stud. Mycol. 96, 1–16. doi: 10.1016/j.simyco.2020.01.001 32165986 PMC7056724

[B137] Wong T. K. F. Ly-Trong N. Ren H. Demotte P. Baños H. Roger A. J. . (2026). IQ-TREE 3: phylogenomic inference software using complex evolutionary models. Mol. Biol. Evol.. 43 (5), msag117. doi: 10.1093/molbev/msag117 PMC13191116 42085559

[B138] Wu J. Dai T. Wang X. Hyde K. D. (2025). Molecular detection methods for forest pathogens. Crit. Rev. Plant Sci. 44 (2), 1–23. doi: 10.1080/07352689.2025.248977637339054

[B139] Xie J. Chen Y. Cai G. Zhang Y. (2023). Tree visualization by one table (tvBOT): a web application for visualizing, modifying and annotating phylogenetic trees. Nucleic Acids Res. 51, W587–W592. doi: 10.1093/nar/gkad359 37144476 PMC10320113

[B140] Yang Z. (2007). PAML 4: phylogenetic analysis by maximum likelihood. Mol. Biol. Evol. 24, 1586–1591. doi: 10.1093/molbev/msm088 17483113

[B141] Yang Y. Xu Y. Wang L. Jiang Q. Q. Su J. Q. Li R. . (2025). Multigene phylogeny of seven wood-inhabiting fungal orders in Basidiomycota, and proposal of a new genus and thirteen new species. Mycosphere 16, 245–295. doi: 10.5943/mycosphere/16/1/4

[B142] Yin Y. Mao X. Yang J. Chen X. Mao F. Xu Y. (2012). dbCAN: a web resource for automated carbohydrate-active enzyme annotation. Nucleic Acids Res. 40, W445–W451. doi: 10.1093/nar/gks479 22645317 PMC3394287

