# Supplementary material for: Unearthing a fungal giant: Dianjunaceae fam. nov., a novel Paleocene lineage of Xylariales harboring Dianjunus rex gen. et sp. nov
Source: Front Fungal Biol. 2026 Sep 29;7:1940464. doi: 10.3389/ffunb.2026.1940464 (PMC13657967; doi:10.3389/ffunb.2026.1940464)
Supplement: Supplementary file 1 [file SupplementaryFile1.docx]

Tables for the BLAST result with ITS, LSU, rpb2, tub2 with five parameters

For LSU

For ITS

For RPB2

For TUB2

**Figure 1.** Phylogenetic tree inferred from maximum likelihood analysis of the ITS sequence alignment contain taxa mostly related to *Dianjunus rex*. Nodes are labelled with maximum likelihood bootstrap support values ≥50%, and Bayesian posterior probabilities ≥0.90. The tree was rooted with *Amphibambusa bambusicola* (MFLUCC 11-0617) and *Cainia graminis* (MFLUCC 15-0540).
